# Ultrathin Membrane With Ion‐Specific Gating Enables Interfacial Decoupling of Conductivity–Selectivity for Aqueous Flow Batteries

**DOI:** 10.1002/anie.2958917

**Published:** 2026-08-22

**Authors:** Di Mu, Yu Xiong, Feiran Wang, Jiafeng Lei, Fei Ai, Jun Fan, Yi‐Chun Lu

**Affiliations:** ^1^ Electrochemical Energy and Interfaces Laboratory Department of Mechanical and Automation Engineering The Chinese University of Hong Kong Hong Kong SAR China; ^2^ Department of Materials Science and Engineering City University of Hong Kong Hong Kong SAR China

**Keywords:** chemisorption, conductivity–selectivity trade‐off, flow batteries, membranes, interface

## Abstract

Ion‐exchange membranes (IEMs) are essential for efficient and durable flow batteries, yet their inherent conductivity–selectivity trade‐off severely limits flow battery performance. Most conventional strategies to improve selectivity rely on regulating ion diffusion within ion‐exchange channels through size exclusion and electrostatic repulsion, inevitably compromising ionic conductivity. Here we shift the paradigm to ion partitioning at the membrane–electrolyte interface by establishing ion‐specific gating that selectively intercepts active species while preserving rapid charge carrier transport. The developed ultrathin (29 ± 2 µm) polar oxygen‐vacancy‐enhanced (POVE) membrane suppresses polysulfide crossover through strong chemisorption and a strengthened hydration layer, delivering over 3.4 times the selectivity of the base membrane at comparable conductivity and over 24.8 times the selectivity of commercial Nafion membranes. In the polysulfide–ferrocyanide flow battery, it achieved 82% energy efficiency at 40 mA cm^−2^ and sustained over 2000 cycles with negligible capacity decay, far exceeding 286 cycles for the base membrane and < 100 cycles for Nafion. This work decouples conductivity from selectivity via a scalable interfacial ion‐specific gating strategy, offering a transformative framework for developing affordable, high‐performance IEMs.

## Graphical abstract

An interfacial ion‐specific gating strategy shifts membrane design from ion diffusion within ion‐exchange channels to ion partitioning at the membrane–electrolyte interface, thereby decoupling selectivity from conductivity. The strategy underscores the pivotal yet underexplored role of the membrane–electrolyte interface, providing a broadly applicable framework for high‐performance membranes.

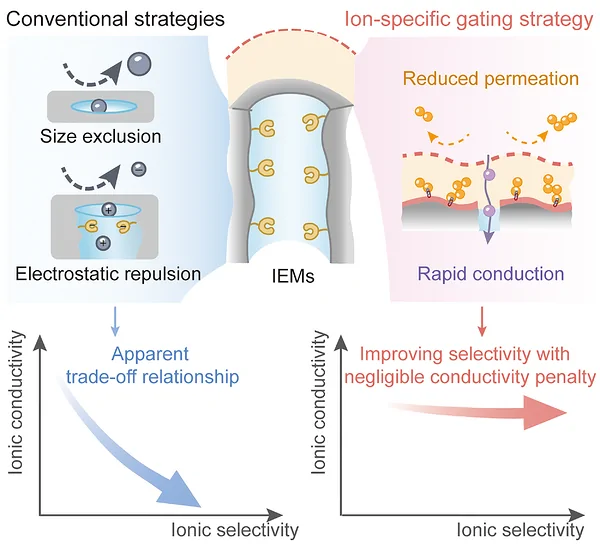

## 1 Introduction

Ion‐exchange membranes (IEMs) are critical components in a broad array of electrochemical systems, including flow batteries [1, 2], fuel cells [3, 4], seawater desalination [5], and water electrolysis [6]. Among these applications, flow batteries impose one of the most demanding requirements on IEMs, necessitating efficient conduction of charge carriers while rigorously blocking crossover of active species. In most flow battery systems, IEMs are the key components that determine the energy efficiency and cycle life, such as polysulfide‐based flow batteries (PBFBs) and vanadium flow batteries (VFBs) [7, 8]. However, a persistent and fundamental challenge of IEMs—the trade‐off between ionic conductivity and selectivity [9, 10]—has long hindered the commercial deployment of flow batteries. For instance, PBFBs possess extremely low chemical costs owing to the high earth abundance of sulfur, while the commercial membrane (e.g., Nafion) fails to provide sufficient potassium ion (K^+^) conductivity and effectively block small (∼9Å) polysulfide anions (S_x_^2−^) crossover [11, 12, 13], leading to low energy efficiency (< 70% at 40 mA cm^−2^) and poor cycle life (< 100 cycles) [14]. Therefore, overcoming the fundamental conductivity–selectivity trade‐off in IEMs is critical to realizing the flow battery's full potential as a low‐cost, durable grid‐scale storage technology.

Ion transport through IEMs encompasses two key processes: ion partitioning at the membrane–electrolyte interface and subsequent diffusion within ion‐exchange channels (Figure 1a) [15]. Conventional strategies to improve ionic selectivity of IEMs primarily focus on engineering the bulk membrane matrix to suppress active species crossover through structural densification of the polymer network and prolongation of tortuous ion transport pathways. Typical approaches include chemical crosslinking, polymer blending, and filler incorporation [16, 17, 18]. For example, incorporating triple tertiary amine‐grafted poly(2,6‐dimethyl‐1,4‐phenylene oxide) (PPO‐TTA) with sulfonated poly(ether ether ketone) (SPEEK) effectively mitigates vanadium ions crossover [17]. Ye et al. reported a hybrid membrane with superhydrophilic TiO_2_ nanotubes in Nafion 212 (N212) matrix, which showed higher coulombic efficiency and improved cycling stability  than the N212 membrane in VFBs [19]. Another charge‐reinforced selective membrane was applied in PBFBs, which significantly suppressed notorious polysulfide shuttling and extended the cycle life by establishing an enhanced electrostatic repulsion layer and reducing the diameter of the ion transport channels [20]. However, these modification approaches primarily regulate the ion diffusion within ion‐exchange channels through nonspecific mechanisms, like size exclusion and electrostatic repulsion (Figure 1b, left), restricting both active species and charge carriers. As a result, efforts to mitigate crossover inevitably impede the very conduction passways for charge carriers, increasing the area‐specific resistance (ASR) of IEMs and amplifying ohmic losses in flow battery applications. Such methods remain constrained by the conductivity–selectivity trade‐off, delivering limited improvements in overall battery performance (Figure 1c).

**FIGURE 1:**
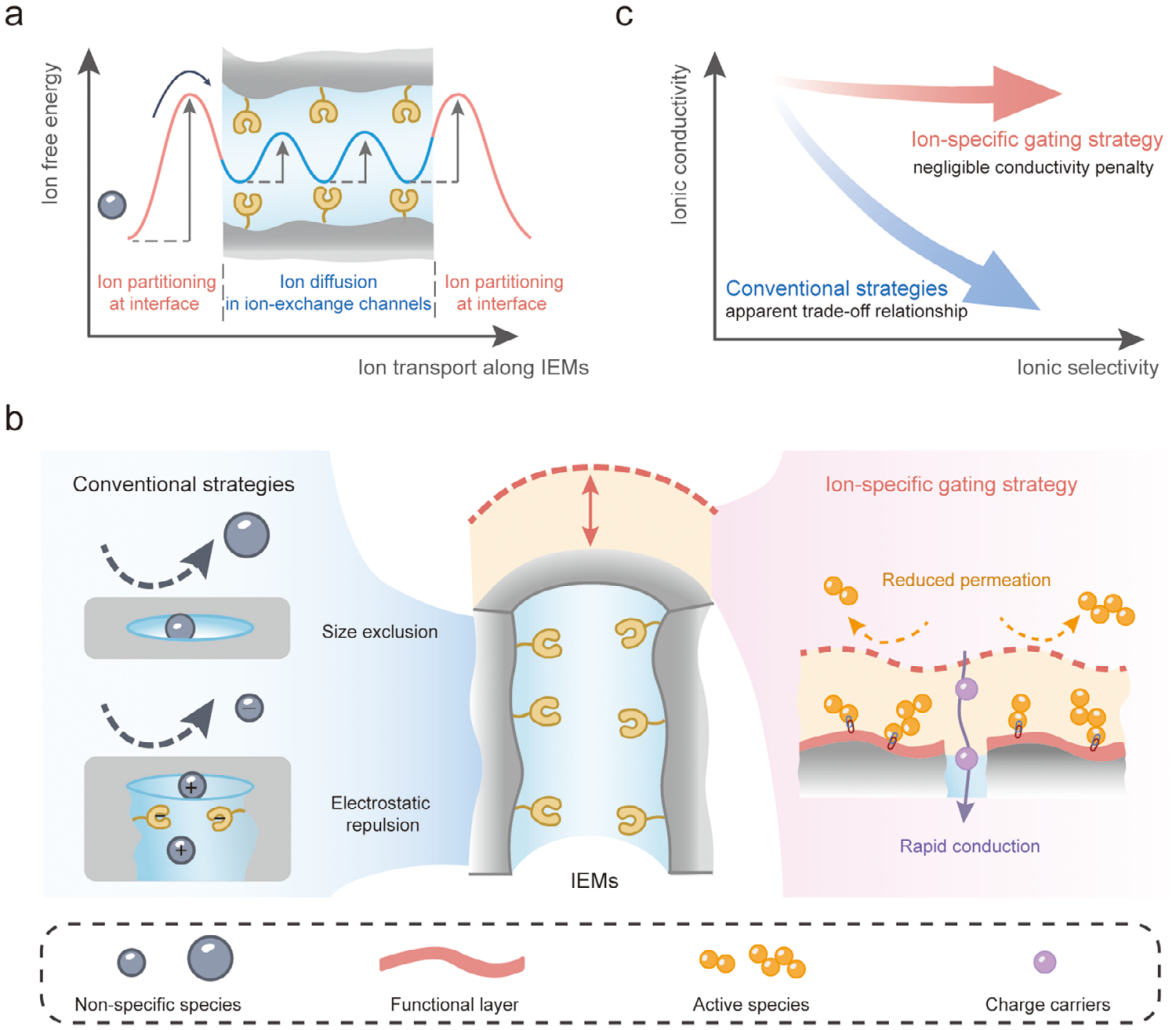
Conductivity–selectivity trade‐off and different design strategies for IEMs. (a) Schematic illustration of the free energy landscape of ion transport through IEMs; (b) Comparison of strategies for enhancing selectivity in IEMs: conventional strategies regulate ion diffusion within ion‐exchange channels (left), and our ion‐specific gating strategy selectively blocks redox‐active species at the membrane–electrolyte interface, preserving rapid conduction of charge carriers (right); (c) Different conductivity–selectivity relationships between conventional strategies and interfacial ion‐specific gating strategy.

Shifting the paradigm from nonspecific bulk‐phase constraints to precise interfacial control, where ion partitioning predominantly occurs, represents a pivotal yet underexplored direction that holds promise for decoupling ionic selectivity from conductivity in IEMs. Biological ion channels provide a compelling blueprint: they achieve rapid transport of target ions with exceptional ionic selectivity through specific chemical interactions at binding sites localized at the membrane surface [21, 22]. Inspired by this natural paradigm, we developed an interfacial ion‐specific gating strategy that precisely targets the membrane–electrolyte interface to selectively intercept active species before they penetrate the membrane's internal transport network, while allowing unimpeded conduction of charge carriers (Figure 1b, right). Specifically, we fabricated an ultrathin (29 ± 2 µm) polar oxygen‐vacancy‐enhanced (POVE) membrane by coating Magnéli‐phase titanium suboxide (Ti_4_O_7_) layers onto a low‐cost and scalable SPEEK base substrate. The abundant oxygen vacancies in Ti_4_O_7_ [23, 24, 25] promoted strong polysulfide adsorption and strengthened the interfacial hydration layer, effectively blocking crossover while minimally impeding K^+^ transport. Consequently, this POVE membrane achieved over 3.4 times the selectivity of the base SPEEK membrane with comparable K^+^ conductivity, and over 24.8 times the selectivity of commercial Nafion membranes. In the polysulfide–ferrocyanide (S–Fe) flow battery, the POVE membrane delivered an energy efficiency of 82% at 40 mA cm^−2^ and sustained over 2,000 cycles with negligible capacity decay, far exceeding 286 cycles for the SPEEK base membrane and < 100 cycles for Nafion. At an estimated cost of 22.6–25.8 US$ m^−2^, which is less than one‐fiftieth that of Nafion 117 (N117) [20, 26, 27, 28] on an average cost basis, the strategy not only decouples the longstanding conductivity–selectivity trade‐off but also offers a scalable route to affordable and high‐performance IEMs for large‐scale flow battery deployment.

## 2 Results and Discussion

### 2.1 Macroscopic Physicochemical Properties Highlight Interfacial Decoupling of Conductivity and Selectivity

Ion transport through IEMs encounters interfacial ion partitioning, where ions rearrange and redistribute energy, forming an electric double layer at the membrane–electrolyte interface; subsequently, these ions migrate through inner hydrated nanochannels, traversing a heterogeneous free energy landscape composed of multiple local barriers [15]. Ionic selectivity arises from differences in the overall free energy profiles governing partitioning and transport for various species. However, in traditional IEMs, the high ionic strength and chemical complexity of flow battery electrolytes weaken interfacial discrimination, and the excessive swelling of hydrated nanochannels inevitably conduct charge carriers along with active species through the shared transport pathways, thereby coupling conductivity with selectivity. Our interfacial ion‐specific gating strategy addresses this by engineering a tailored interfacial layer that preferentially anchors active species while adding negligible penalties to charge carriers conduction, thus decoupling the two properties.

We fabricated the ultrathin (29 ± 2 µm) POVE membrane using low‐cost, easily prepared SPEEK as the base membrane and coating it with Magnéli‐phase Ti_4_O_7_, whose oxygen vacancies confer strong polysulfide affinity [23, 24, 25]. Bare SPEEK membrane with comparable thickness, carbon‐based Ketjen black‐decorated SPEEK (S@KB) membrane, and commonly used N212 and N117 are included for comparison (Figure S1). The cross‐sectional scanning electron microscopy (SEM) images and digital photographs revealed uniform, dense morphologies for Nafion series and SPEEK base membranes, while S@KB and POVE membranes exhibited sandwich architectures with functional layers coating on both sides (Figure 2a). Energy‐dispersive x‐ray spectroscopy (EDS) mapping further confirmed the elemental distributions of these ultrathin layers (Figures S2 and S3).

**FIGURE 2:**
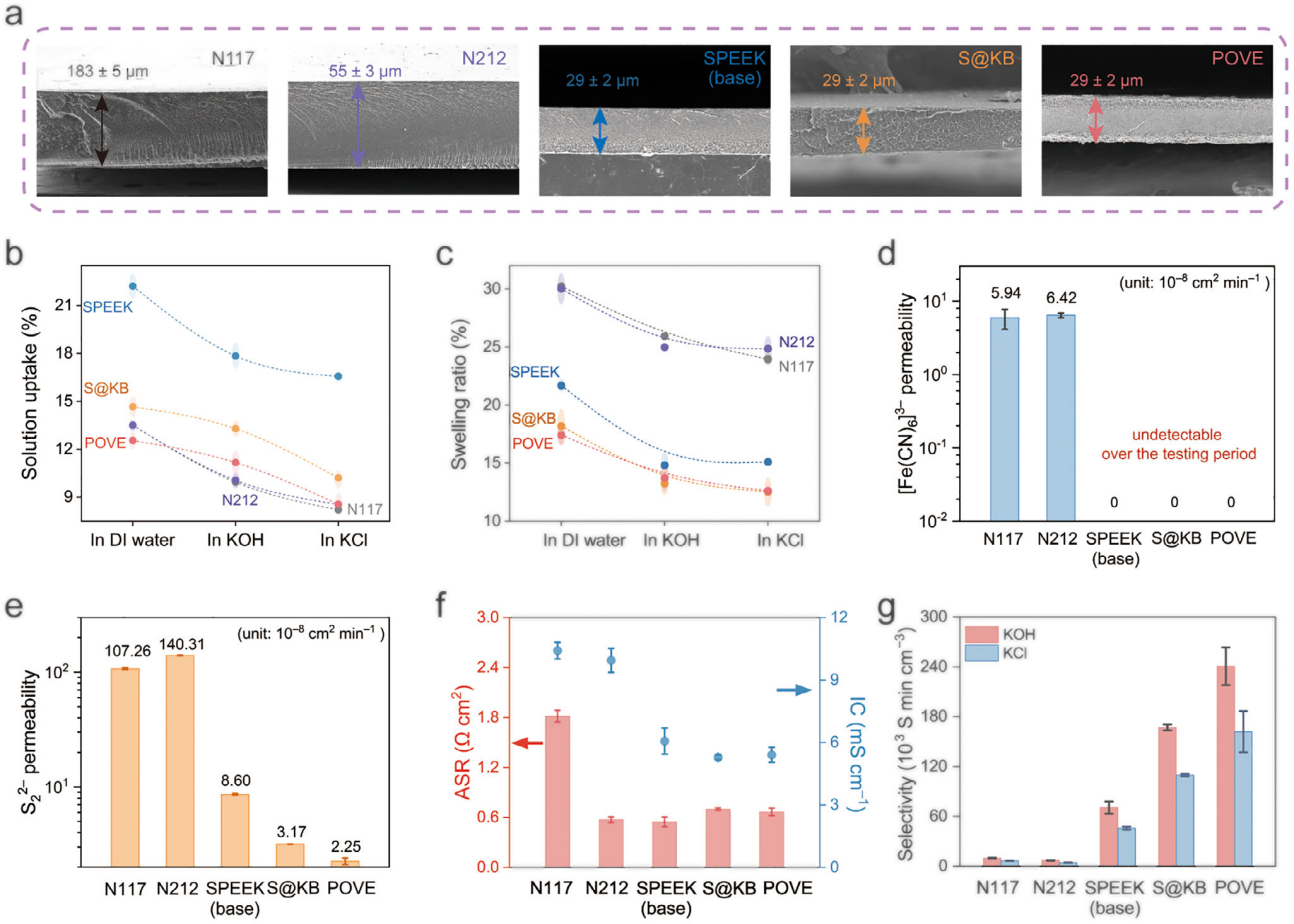
Macroscopic physicochemical properties of different IEMs. (a) Cross‐sectional SEM images; (b) Solution uptake in various aqueous solutions; (c) Swelling ratios in various aqueous solutions; (d) Permeability of [Fe(CN)_6_]^3−^; (e) Permeability of S_2_^2−^; (f) Area‐specific resistance (ASR) and ionic conductivity (IC) in 1 M KOH solution; (g) K^+^/S_2_^2−^ selectivity. The shallow ranges and error bars represent the standard deviation from at least three independent measurements for each membrane and condition.

Solution uptake is vital for forming hydrated, interconnected ion‐transport pathways, but excessive swelling can expand channels and reduce ionic selectivity. We noted that all membranes showed lower electrolyte uptake and swelling ratios in 1 M KOH and 1 M KCl aqueous solutions compared to deionized (DI) water (Figure 2b,c, and Table S1), and their swelling ratios decreased progressively with increasing saline/alkaline concentrations (Figure S4). This concentration‐dependent deswelling could be attributed to the reduced osmotic pressure and enhanced screening of the fixed sulfonate charges at higher external solution concentrations, which together suppress water uptake and expansion of the hydrated ionic domains [14]. N212 and N117 showed relatively low uptake but high swelling ratios in DI water, 1 M KOH, and 1 M KCl, attributable to their phase‐separated structure from rigid hydrophobic backbones and pendant hydrophilic side chains [14, 15, 29]. In contrast, the SPEEK membrane exhibited higher solution uptake with moderate swelling ratios. The S@KB and POVE membranes further showed slightly lower swelling ratios compared to the SPEEK substrate membrane while largely retaining the internal water channel density (Figure S5), suggesting that surface coatings limited electrolyte‐induced dimensional expansion without substantially disrupting the ion‐transport structures within the SPEEK substrate.

Ionic permeability was quantified using UV–visible spectroscopy in an H‐cell configuration (Figures S6 and S7). For posolytes, ferricyanide ions ([Fe(CN)_6_]^3−^) and ferrocyanide ions ([Fe(CN)_6_]^4−^) permeated rapidly through N212 (55 ± 3 µm, post‐preparation), and tripling the membrane thickness to N117 (183 ± 5 µm, post‐preparation) suppressed the accumulated concentration to about one‐third (Figure S8a,b). While Nafion series (including N212 and N117) still showed markedly higher ionic permeability than the ultrathin SPEEK‐based IEMs (including SPEEK, S@KB, and POVE), in which the crossover of [Fe(CN)_6_]^3−^ and [Fe(CN)_6_]^4−^ remained undetectable over the testing period (Figure 2d and Figure S9). These results underscored the excellent blocking ability of our prepared ultrathin SPEEK‐based membranes toward active materials in posolyte. In terms of S_2_^2−^, a similar thickness‐dependent permeation trend was also observed in conventional Nafion series membranes: the thinner N212 exhibited nearly three times the flux of N117 and failed prematurely due to severe crossover (Figure S8c). However, despite being only one‐sixth as thick as N117, the SPEEK membrane reduced permeation to one‐third that of N117. Incorporation of ion‐specific interfacial layers further suppressed S_2_^2−^ crossover, with S@KB and POVE membranes reducing permeation to about half and one‐third of the SPEEK substrate membrane, respectively, at comparable thickness. Notably, the POVE membrane showed the smallest S_2_^2−^ permeation rate (2.25 × 10^−8^ cm^2^ min^−1^) among all membranes, substantially lower than those of Nafion membranes (107.26 × 10^−8^ cm^2^ min^−1^ for N117 and 140.31 × 10^−8^ cm^2^ min^−1^ for N212; Figure 2e). The relative permeation rates of S_4_^2−^ across different membranes also followed the same ranking order as S_2_^2−^ (Figure S8d). Among all tested ion species, S_2_^2−^ exhibited the highest permeability, which markedly exceeded that of S_4_^2−^ and was about an order of magnitude higher than those of [Fe(CN)_6_]^3−^ and [Fe(CN)_6_]^4−^ (Figure S9 and Table S2), making it a direct and sensitive metric for evaluating polysulfide‐blocking efficacy of IEMs.

Area‐specific resistance (ASR) and ionic conductivity (IC) were measured via electrochemical impedance spectroscopy (EIS) in 1 M KOH and 1 M KCl, respectively (Figure 2f and Figures S10 and S11). Although the ionic conductivity of the SPEEK base substrate (6.0 mS cm^−1^ in KOH) was slightly lower compared to Nafion membranes (9.9 mS cm^−1^ for N212 and 10.4 mS cm^−1^ for N117 in KOH), its markedly reduced thickness enabled a comparable area‐specific resistance to that of N212 (0.55 Ω cm^2^ for N212 versus 0.57 Ω cm^2^ for SPEEK in KOH) and only one‐third that of N117 (1.82 Ω cm^2^ in KOH). The interfacial coatings introduced marginal area‐specific resistance penalties, maintaining the intrinsic low‐resistance advantages of the ultrathin SPEEK membrane. The ionic conductivities of S@KB and POVE membranes further confirmed the negligible impact on K^+^ transport. Consequently, the K^+^/S_2_^2−^ selectivity (Figure 2g and Figure S12; Table S3) was substantially enhanced in the ultrathin ion‐specific gating membranes: the POVE membrane reached 2.4 × 10^5^ S min cm^−3^ in KOH and 1.6 × 10^5^ S min cm^−3^ in KCl, corresponding to over 3.4 times the ionic selectivity compared to the SPEEK base membrane while maintaining near‐identical conductivity, and over 24.8 times the ionic selectivity compared to widely used Nafion membranes.

Long‐term stability is essential for the practical deployment of flow batteries [30, 31]. The prepared membranes exhibited balanced mechanical rigidity, superior thermal integrity across operational temperatures, and robust chemical stability after 60 days in posolytes and negolytes (Figures S13–S16 and Note S1), supporting their reliability for extended cycling in flow batteries.

### 2.2 Microscopic Mechanisms Reveal the Role of Strong Adsorption and Strengthened Hydration Layer in Gating

To elucidate how interfacial ion‐specific gating achieves this decoupling function, we probed microscopic structures of various IEMs. Small‐angle x‐ray scattering (SAXS) of fully hydrated membranes showed continuous ion‐exchange channels in all SPEEK‐based variants, with nearly unchanged characteristic peaks at 3.23–3.07 nm^−1^ (Figure S17). This confirmed that our surface modifications leave the internal nanostructure largely intact, attributing the observed conductivity–selectivity decoupling to enhanced ion‐specific gating at the interface rather than bulk‐phase alterations. Figure 3a schematically illustrates the interfacial differences between S@KB and POVE membranes. The S@KB membrane relies on weak van der Waals–driven physisorption between porous carbon and polar polysulfide ions at the outer Helmholtz plane. In contrast, the Magnéli‐phase Ti_4_O_7_ on the POVE membrane promotes strong chemisorption at the inner Helmholtz plane [23, 24, 25], coupled with a strengthened hydration layer that further suppresses polysulfide crossover.

**FIGURE 3:**
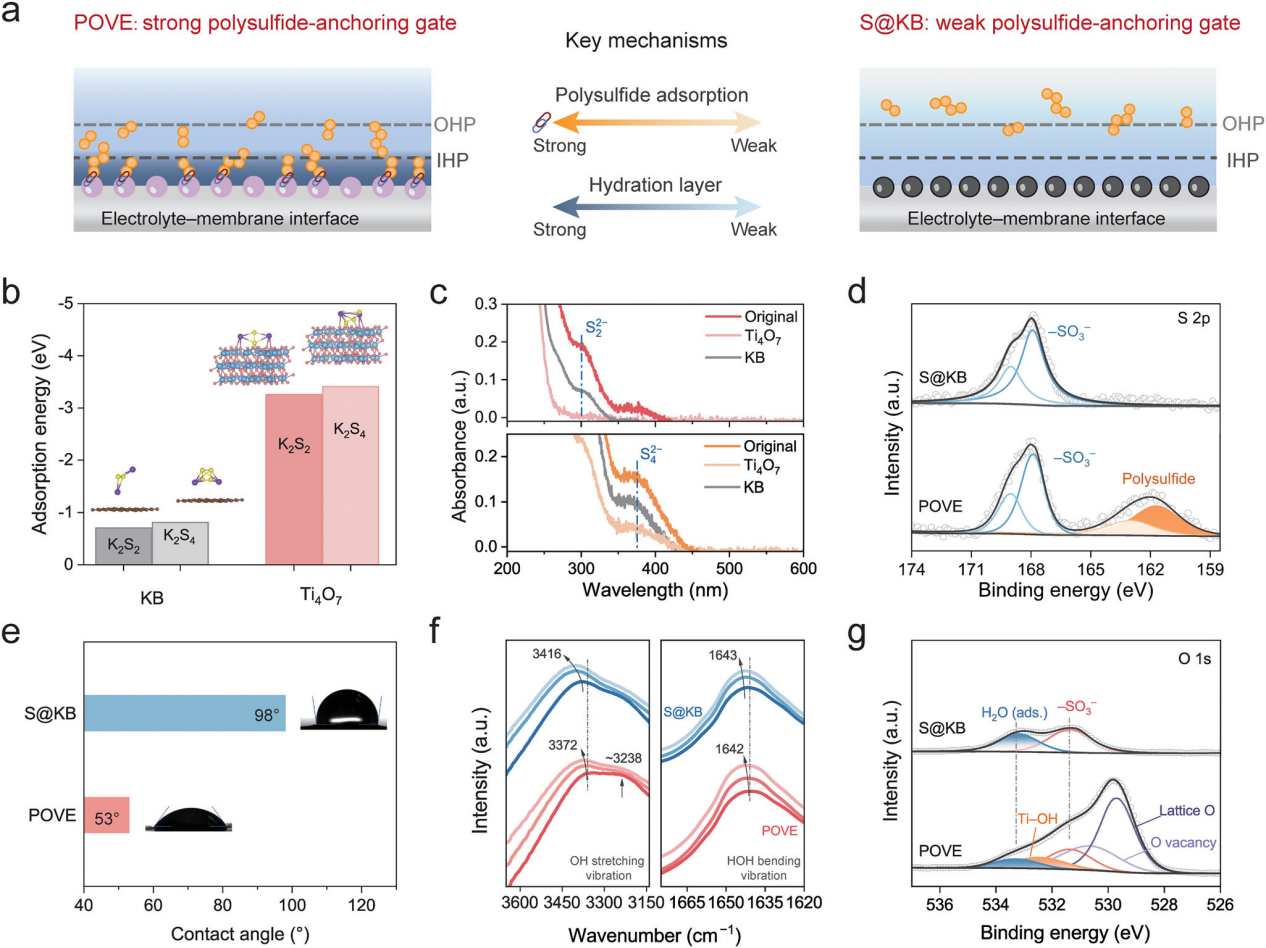
Microscopic comparison of ion‐specific gating membranes with different polysulfide adsorption and hydration layer architectures. (a) Comparison of different electrolyte–membrane interfaces between POVE and S@KB membranes (purple particles for Ti_4_O_7_, black particles for KB and yellow particles for S_x_^2−^); (b) Density functional theory (DFT) calculations of polysulfide adsorption energies of Ti_4_O_7_ and KB; (c) UV–visible spectra of polysulfide ions before (original profile) and after soaking with Ti_4_O_7_ and KB, respectively; (d) XPS S 2p high‐resolution spectra of S@KB and POVE membrane after soaking in polysulfide solution; (e) The water contact angles of S@KB and POVE membranes; (f) ATR–FTIR spectra of fully hydrated S@KB and POVE membranes during dehydration for 0, 6, and 12 min (from darkest to lightest color); (g) XPS O 1s high‐resolution spectra of S@KB and POVE membrane after soaking in polysulfide solution.

Ti_4_O_7_ belongs to the sub‐stoichiometric titanium oxides, bridging TiO_2_ and Ti_2_O_3_ with oxygen‐vacancy‐driven shear planes of Ti–O octahedra (Figure S18). This endows Ti_4_O_7_ with metallic conductivity comparable to that of carbon‐based materials (Figure S19) [32, 33], which is crucial for low‐resistance gating of IEMs. Theoretical calculations confirmed strong chemical binding between Ti_4_O_7_ and polysulfides. Ti_4_O_7_ showed much stronger polysulfide adsorption than KB, with adsorption energies of −3.25 and −3.41 eV for K_2_S_2_ and K_2_S_4_, compared with −0.71 and −0.81 eV for KB, respectively (Figure 3b and Figure S20). The corresponding shorter substrate–S distances on Ti_4_O_7_ (2.45 and 2.57 Å) than KB (4.64 and 5.92 Å) further confirmed the stronger interfacial interactions (Table S4 and Note S2). Moreover, the high diffusion energy barriers restricted the diffusion of adsorbed polysulfides, and *ab initio* molecular dynamics (AIMD) simulations demonstrated the stable polysulfide binding under hydrated conditions (Figures S21–S23 and Note S3). Consistent with theoretical calculations, experimental adsorption tests also demonstrated strong polysulfide affinity of Ti_4_O_7_: the characteristic UV–visible peaks for S_2_^2−^ (300 nm) and S_4_^2−^ (375 nm) [20] diminished markedly with Ti_4_O_7_, but only slightly attenuated with KB (Figure 3c). These results can be attributed to the different adsorption mechanisms between functional materials and polysulfides. Unlike KB's surface‐area‐dependent physisorption, polysulfide adsorption by Ti_4_O_7_ arises from chemical binding at abundant active sites [23, 34]. High‐resolution x‐ray photoelectron spectroscopy (XPS) S 2p spectra of the POVE membrane after polysulfide immersion showed chemisorption peaks at 161.9 and 162.8 eV, while absent in the S@KB membrane (only sulfonate group signals at 167.9 and 169 eV; Figure 3d) [35, 36, 37]. The strong interaction facilitates the rapid immobilization of polysulfide ions, preventing further crossover.

The strengthened hydration layer on the POVE membrane adds a second gating mechanism. Ions partially dehydrate and reorganize hydration shells at the interface, with associated energy barriers sensitive to ionic size, hydration free energy and surface hydrophilicity [38, 39, 40, 41]. Compared with K^+^, S_x_^2−^ with larger hydrated dimensions are expected to experience overall higher penalties for their structural rearrangement and partial dehydration when traversing the highly structured hydration layer, thereby further lowering the possibility of S_x_^2−^ penetrating through the membrane [14, 42]. As shown in Figure 3e, the contact angle was only 53° for the POVE membrane, reflecting the hydrophilic nature of Ti_4_O_7_; whereas an increased value of 98° for the S@KB membrane, indicating its surface hydrophobicity. Compared with S@KB membrane, attenuated total reflectance Fourier–transform infrared (ATR–FTIR) spectra of fully hydrated POVE membrane exhibited pronounced redshifts in both the O–H stretching and H–O–H bending vibration (Figure 3f). Besides, a prominent increased peak appeared at 3238 cm^−1^ in the POVE membrane. During the dehydration process, the POVE membrane showed significantly smaller blueshifts in both characteristic peaks, especially in the O–H stretching vibration, which were only an 8 cm^−1^ blueshift to 3372 cm^−1^ for POVE while a 38 cm^−1^ blueshift to 3416 cm^−1^ for the S@KB membrane. These spectral changes indicated a strengthened and stable hydrogen‐bond network of the POVE membrane, consistent with the formation of a more structured hydration layer. XPS O 1s spectra also showed Ti−OH peak, which corroborates the stronger interfacial hydration environment in the POVE membrane (Figure 3g). The POVE's more negative surface charge in negolyte also enhanced electrostatic repulsion at the shear plane (Figure S24).

### 2.3 Efficient and Durable Performance in Aqueous S–Fe Flow Batteries Demonstrates Practical Impact

We evaluated the membranes in S–Fe flow batteries employing S_2_^2−^/S_4_^2−^ (−0.46 V versus ***V***_**SHE**_) as the negolyte redox couple and [Fe(CN)_6_]^4−^/[Fe(CN)_6_]^3−^ (0.45 V versus ***V***_**SHE**_) as the posolyte redox couple (Figure 4a) [43, 44, 45]. S–Fe flow battery with N117 displayed nearly twice the resistance than that of SPEEK‐based membranes (Figure 4b and Figure S25). As a result, N117 showed significantly high overpotentials across all current densities, limiting the maximum stable operation to 120 mA cm^−2^ (Figure 4c). Beyond this, the discharge capacity collapsed mainly due to the rapid crossover‐induced sulfur accumulation and resistance‐driven failure (Figure S26). In contrast, the S–Fe flow batteries equipped with our prepared S@KB and POVE membranes sustained up to 200 mA cm^−2^, with discharge capacities consistently exceeding N117 and N212 from 20 to 200 mA cm^−2^ (Figure 4d and Figure S27). The flow batteries assembled with S@KB and POVE membranes reached the energy efficiency of 81% and 82% at 40 mA cm^−2^, respectively, which was comparable to the SPEEK base membrane, versus only 69% for N117 (Figure S28). Furthermore, power densities of the S–Fe flow batteries improved markedly (Figure S29): N117 peaked at 63.5 mW cm^−2^ (@130 mA cm^−2^), while POVE membrane achieved 109.4 mW cm^−2^ (@210 mA cm^−2^) and S@KB membrane reached 104.6 mW cm^−2^ (@200 mA cm^−2^), respectively. The POVE membrane's edge at higher densities than the S@KB membrane is likely attributable to stronger chemisorption anchoring polysulfides more effectively under harsh conditions, thereby extending the operational range. The open‐circuit voltage (OCV) decay test reflects the overall crossover behavior under continuous‐flow conditions. The flow battery with N117 sustained only 153 h before a rapid voltage drop, and N212 exhibited even faster voltage decay, failing within just 46 h (Figure 4e and Figure S30). These results reflected the severe polysulfide shuttling and thickness dependence in both Nafion membranes. In contrast, the S–Fe flow battery with the POVE membrane maintained high voltage for over 600 h, confirming its superior crossover‐blocking ability compared to the SPEEK base membrane (289 h). As a result, the flow battery with POVE membrane delivered exceptional cycling stability, sustaining over 2000 cycles with over 99.9997% capacity retention per cycle (Figure 4f and Figure S31), far outperforming batteries using SPEEK base substrate (286 cycles) and S@KB membrane (∼1000 cycles). By contrast, Nafion membranes failed much earlier, which was 98 cycles for N117 and 61 cycles for N212. We further explored the applicability of the POVE membrane to other polysulfide‐based flow battery systems, such as polysulfide‐polyiodide (S–I) flow battery (Figure S32). Owing to its low membrane resistance and effective suppression of polysulfide crossover, the POVE membrane enabled reversible operation over current densities ranging from 20 to 80 mA cm^−2^ and showed stable cycling performance over 250 cycles at 40 mA cm^−2^, with an average coulombic efficiency exceeding 99.6% and an average energy efficiency over 82.6%. Post‐cycling characterizations (Figures S33–S36 and Note S4) showed the minimal structural degradation and limited surface deposition of the POVE membrane, supporting the long‐term durability of the ultrathin ion‐specific gating IEMs.

**FIGURE 4:**
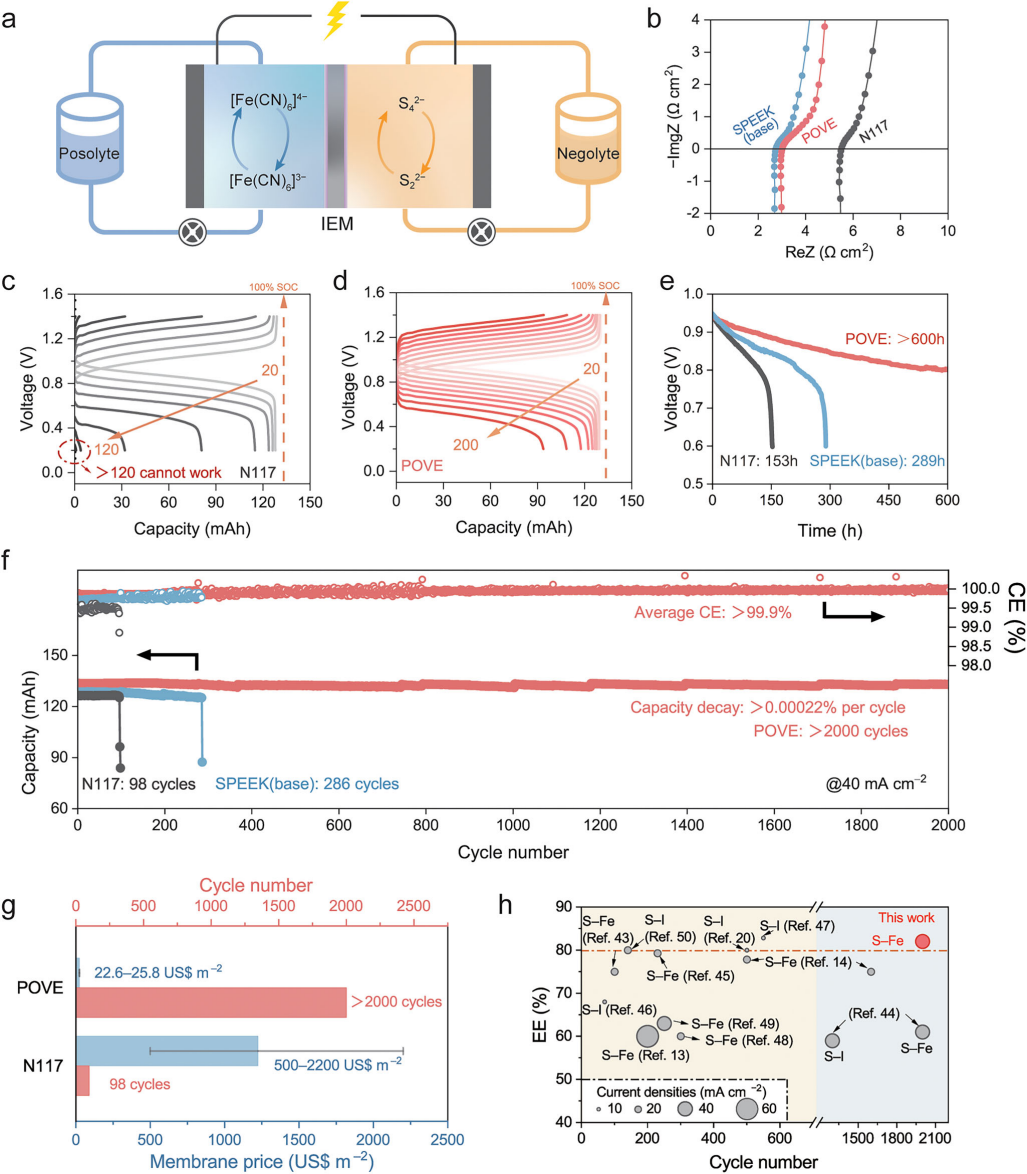
Efficient and durable aqueous S–Fe flow battery performance. (a) Schematic illustration of aqueous S–Fe flow battery assembled with IEM; (b) Nyquist plots of various IEMs by EIS test; (c) Voltage–current profiles of the S–Fe flow battery with N117 membrane, the color change indicates increasing current density from 20 mA cm^−2^ (lightest) to 200 mA cm^−2^ (darkest) with a 20 mA cm^−2^ interval; (d) Voltage−current profiles of the S–Fe flow battery with POVE membrane, the color change indicates increasing current density from 20 mA cm^−2^ (lightest) to 200 mA cm^−2^ (darkest) with a 20 mA cm^−2^ interval; (e) Open‐circuit voltage (OCV) curves of various IEMs; (f) Long‐term cycling stability of various IEMs under the current density of @40 mA cm^−2^; (g) The comparison of the membrane price and cycle number of N117 and POVE membranes (error bars represent the price range of IEMs); (h) Cycling performance of the S–Fe flow battery with POVE membrane compared with recently reported PBFBs [13, 14, 20, 43, 44, 45, 46, 47, 48, 49, 50].

A cost–performance comparison emphasizes the impact of the ultrathin POVE membrane (Figure 4g, Tables S5 and S6). With a production cost of 22.6–25.8 US$ m^−2^, the POVE membrane enabled stable operation for over 2000 cycles. In stark contrast, N117 membranes (500−2200 US$ m^−2^) [20, 26, 27, 28], with a cost of over 50‐fold higher on an average basis, failed the operation after just 98 cycles. Figure 4h and Table S7 compare the S–Fe flow battery performance using the POVE membrane with that of other recently reported PBFBs. The POVE membrane exhibited a rare combination of high energy efficiency and long‐term cycling stability, underscoring the effectiveness of this interfacial ion‐specific gating strategy in overcoming membrane limitations of PBFBs. The strategy highlights the pivotal role of the membrane–electrolyte interface and offers a scalable route to affordable IEMs in diverse energy systems, facilitating grid‐scale energy storage for renewable energy integration.

## 3 Conclusion

We have shown that shifting the design from nonspecific regulation within bulk‐phase ion‐exchange channels to ion partition at the membrane–electrolyte interface offers a promising design route to addressing the longstanding conductivity–selectivity trade‐off in IEMs. An interfacial ion‐specific gating strategy was rigorously validated in PBFBs, where low K^+^/S_2_^2−^selectivity led to sulfur precipitation, membrane degradation, and eventual battery failure. By designing ultrathin (29 ± 2 µm) low‐cost SPEEK‐based membranes with tailored interfacial ion‐specific gating, we achieved precise control over ion‐selective transport with ionic conductivity largely intact. The POVE membrane anchors polysulfides through strong chemisorption and a strengthened hydration layer, outperforming the S@KB membrane's weak physisorption as well as its base SPEEK membrane. As a result, the POVE membranes delivered over 3.4 times the K^+^/S_2_^2−^ selectivity of its base membrane with comparable conductivity, and over 24.8 times K^+^/S_2_^2−^ selectivity relative to commercial Nafion membranes. In the S–Fe flow battery, the POVE membrane enabled stable operation at current densities up to 200 mA cm^−2^, delivered approximately 82% energy efficiency at 40 mA cm^−2^, and sustained over 2000 cycles with negligible capacity fade (∼1000 cycles for S@KB and <100 cycles for both N117 and N212). The POVE achieved an estimated cost of 22.6–25.8 US$ m^−2^, illustrating the practicality of this interfacial ion‐specific gating strategy that not only breaks the selectivity–conductivity trade‐off but also offers a simple, effective, and broadly applicable framework for high‐performance, low‐cost IEMs. Beyond this detailed example in PBFBs, the strategy also demonstrates the pivotal yet underexplored role of the membrane–electrolyte interface, which offers a versatile design route to enhance electrochemical energy storage performance.

## Author Contributions

**Di Mu**: conceptualization, methodology, data curation, investigation, validation, formal analysis, visualization, writing – original draft, writing – review & editing. **Yu Xiong**: methodology, formal analysis, writing – review & editing. **Feiran Wang**: methodology, investigation, writing – review & editing. **Jiafeng Lei**: investigation, methodology, writing – review & editing. **Fei Ai**: methodology, writing – review & editing. **Jun Fan**: methodology, writing – review & editing, funding acquisition. **Yi‐Chun Lu**: conceptualization, writing – review & editing, supervision, funding acquisition, project administration.

## Conflicts of Interest

The authors declare no conflicts of interest.

## Supporting information



Experimental methods and other data that support the findings of this study are available in the accompanying Supporting Information. The authors have cited additional references within the Supporting Information ^[51–66]^.**Supporting File**: anie74329‐sup‐0001‐SuppMat.pdf.

## Data Availability

The data that support the findings of this study are available from the corresponding author upon reasonable request. The experimental results are presented in the supplemental information.
