# Supplementary material for: Ultrathin Membrane With Ion‐Specific Gating Enables Interfacial Decoupling of Conductivity–Selectivity for Aqueous Flow Batteries
Source: Angew Chem Int Ed Engl. 2026 Aug 22;65(42):e2958917. doi: 10.1002/anie.2958917 (PMC13653613; doi:10.1002/anie.2958917)
Supplement: Supplementary file 1 [file ANIE-65-e2958917-s001.pdf]

Supporting Information

Ultrathin Membrane with Ion-Specific Gating Enables Interfacial Decoupling of Conductivity–Selectivity for Aqueous Flow Batteries

Di Mu,[a] Yu Xiong, [b] Feiran Wang,[a] Jiafeng Lei,[a] Fei Ai,[a] Jun Fan, [b] and Yi-Chun Lu*[a]

[a] Electrochemical Energy and Interfaces Laboratory, Department of Mechanical and Automation Engineering, The Chinese University of Hong Kong, Shatin, N.T. 999077, Hong Kong SAR, China.

[b] Department of Materials Science and Engineering, City University of Hong Kong, Hong Kong SAR, China.

*Corresponding Author: Yi-Chun Lu. E-mail: yichunlu@mae.cuhk.edu.hk

This file includes:

Experimental Methods

Supplymentary Figures S1 to S36

Supplymentary Tables S1 to S7

Supplementary Notes 1 to 4

Supplementary References 51 to 66

Experimental Methods

Materials

Potassium (poly) sulfide (K_2S_x, ≥42% K_2S basis, x is determined to be ~2.0), sulfur (S, more than 99.5%), potassium hydroxide (KOH, ≥95 %), potassium chloride (KCl, >99.8%), potassium ferrocyanide ($K_4[Fe(CN)_6]\cdot 3H_2O$, ≥99.5%), potassium ferricyanide ($K_3[Fe(CN)_6]$, ≥99.5%), sodium hydroxide (NaOH, 99.7%), 2-Propanol (IPA, anhydrous, ≥99.5%), N,N-Dimethylformamide (DMF), sulfuric acid (H_2SO_4, 95%–98%), hydrogen peroxide (H_2O_2, 30 wt.% in H_2O), sodium chloride (NaCl, 99.9%) were received from Sigma-Aldrich. Ethanol was received from VWR International S.A.S. Polytetrafluoroethylene (PTFE) and phenolphthalein (pH indicator) were received from Dieckmann. Ketjen black (KB, ECP–600JD) was received from Lion Corporation. Titanium suboxide (Ti_4O_7, ≥96.8%) was bought from Henan Longxing Titanium Industry Technology Co., Ltd. Nafion membranes (Nafion 117 and Nafion 212, Dupont) were received from H2FLOW (Shanghai) Advanced Materials International Trade Co., Ltd. Carbon felts (GFD 4.6EA) were received from SGL Carbon SE. Nickel foam (purity 99.8%, porosity 97.2%, thickness 1.5 mm, areal density 350 ± 20 g m^{-2}) was received from Zhengzhou Jinghong New Energy Technology Co., Ltd. Poly (ether ether ketone) (PEEK) were received from Victrex.

Membrane Preparation

Sulfonated poly (ether ether ketone) (SPEEK) was prepared by sulfonating PEEK (Victrex 450G) with concentrated sulfuric acid at 50 °C for 105 min. The resulting solution was then poured into an ice–water mixture under continuous stirring and washed with deionized (DI) water to neutral pH. Finally, the sulfonated polymer (SPEEK) was dried at 80 °C overnight.

Membranes were prepared by the solution casting method. In a typical procedure, a 7 wt% casting solution was used (DMF as the solvent). The mixture was stirred over 12 h to obtain a homogenous casting solution, which was then cast and dried for 12 h at 100 °C. After cooling to room temperature, the membrane was peeled off and saturated in 1M KOH for 12 h, and flushed with DI water before use.

The functionalized membrane was prepared by spray-coating method. Ti_4O_7 was first washed with ethanol under ultrasonication and centrifuged at 3500 rpm for 1 min. The supernatant was collected and dried for subsequent use. A coating slurry with Ti_4O_7: PTFE = 13:1 (in mass) was dispersed in 2 mL IPA solution; after stirring overnight to maintain a uniform slurry, it was spray-coated onto both sides

of the SPEEK membrane (0.4 mL slurry per side). Afterwards, the membrane was heated at 80 °C in an oven for 8 h and then soaked in 1M KOH for 12 h and flushed with DI water before use. The S@KB membrane was prepared following the same procedure, with the material loading adjusted to ensure an equivalent molar amount to that of Ti_4O_7. Nafion 117 (N117) and Nafion 212 (N212) were prepared according to our previously reported method.[14]

Characterization

Ion exchange capacity (IEC) and degree of sulfonation (DS). The IEC and DS were evaluated using an acid-base titration method. Briefly, the prepared SPEEK membrane was first reactivated in 1M H_2SO_4 (24 h). After being thoroughly washed with excess DI water, the membrane was dried in an oven at 80 °C for 24 h, and the weight was recorded as W_{dry}. Afterwards, the dry membrane was immersed in a saturated NaCl solution for 48 h. The resulting solution was titrated with 0.1 M NaOH solution with phenolphthalein as the pH indicator. The IEC of the polymer was calculated using the following equation:

$$\text{IEC}\left(\text{mmol g}^{-1}\right) = \frac{V_{NaOH} \times C_{NaOH}}{W_{dry}} \tag{1}$$

where V_{NaOH} is the volume (μL) of NaOH solution at the endpoint when phenolphthalein changes to red color; W_{dry} indicates the weight (mg) of dry membrane.

The DS was calculated by the following equation:

$$\text{DS}\ (\%) = \frac{288 \times \text{IEC}}{1000 - 80 \times \text{IEC}} \tag{2}$$

The reactivated DS was 51% in this work.

Morphology. The morphology of membranes was analyzed using scanning electron microscope (SEM) and energy dispersive X-ray spectroscopy (EDS). To observe the cross-section, the membranes were cryo-fractured in liquid nitrogen.

Solution uptake and swelling ratio. Different membranes were fully dehydrated in a vacuum oven at 80 °C for 24 h. The dehydrated membrane was immersed in various solutions (including DI water, 1 M KOH and 1M KCl) at room temperature for 24 h to reach a hydrated state. The solution uptake (ω) was

determined as the weight ratio of absorbed solution to the dehydrated membrane:

$$\omega = \frac{\mathrm{m}_h - \mathrm{m}_d}{\mathrm{m}_d} \times 100\% \tag{4}$$

where m_h and m_d are the mass of hydrated and dehydrated membrane, respectively.

The swelling ratio (φ) was defined as the volume expansion ratio of absorbed solution to dehydrated membrane:

$$\varphi = \frac{\mathrm{V}_h - V_d}{\mathrm{V}_d} \times 100\% \tag{5}$$

where V_h and V_d are the dimensional sizes of hydrated and dehydrated membrane, respectively.

Mechanical, thermal, and chemical stability. The mechanical properties of different hydrated membranes were measured using electro-mechanical universal testing machine (INSTRON 5969). The membranes were cut into strips with dimensions of 1 cm × 5 cm. Thermogravimetric analysis (TGA) was conducted on Mettler TGA2 to evaluate the thermal stability of the membranes. To assess chemical stability, all membrane samples were immersed in different electrolytes for 60 days at room temperature. Following washing with excessive DI water, attenuated total reflectance–Fourier transform infrared (ATR–FTIR, ThermoFisher) spectroscopy was collected in the range of 500–4,000 cm^{-1}.

Small-angle X-ray scattering (SAXS). SAXS was tested on Xenocs Xeuss 2.0 with the detector (Pilatus 3R 300K) and X-ray wavelength of λ=1.54Å. The sample detector distance was fixed at 538 mm. The water cluster size was calculated by $d = {}^{2\pi}/_{q}$ based on Bragg's law.

Ionic conductivity and permeability. Electrochemical impedance spectroscopy (EIS) was used to measure area-specific resistances (ASR) and ionic conductivity (IC) in a static cell assembled with different membranes. The measurements were conducted over a frequency range from 0.2 MHz to 0.1 Hz. The ASR and IC of the membranes were determined using the equations below:

$$\mathrm{ASR} = \mathrm{A} \times (R_w - R_o) \tag{6}$$

$$IC = \frac{L}{A \times (R_w - R_o)} \tag{7}$$

Here, A and L represent the membrane's active area and thickness, R_w and R_o are the resistances of

the static cell with and without the membrane, respectively. Before testing, the membranes were soaked in the individual testing solution for 24 h.

Ionic permeability was evaluated using an H-cell configuration, in which the membrane specimen was securely clamped between feed and permeation chambers. For polysulfide ions permeation tests, the feed side contained 1M K_2S_2 or K_2S_4 in 1M KOH solution, and the permeate compartment was 1M KOH. For ferrocyanide/ferricyanide tests, the feed solution was 0.5 M $K_4[Fe(CN)_6]$ or 0.5 M $K_3[Fe(CN)_6]$ in 1.0 M KCl, and the permeate solution was 1.0 M KCl. Both compartments were filled with 8 mL of solution. Ion concentrations in the permeate side were quantified by UV–Visible Spectrophotometer (ASL SEC2020) at room temperature. The permeability coefficient (D) was calculated using the following equation:

$$V_p \frac{dc_p(t)}{dt} = \frac{AD}{L}\left[c_f - c_p(t)\right] \tag{8}$$

In this formula, c_f and $c_p(t)$ are the concentrations of ions in the feed and permeate sides, V_p is the volume of solution in the permeate side; A and L are the effective area and thickness of the membrane sample. The ferrocyanide/ferricyanide testing was conducted under standard (ambient) conditions. To prevent air-induced polysulfide oxidation, all polysulfide permeation measurements were conducted in the argon-filled glovebox. Aliquots (100 μL) were withdrawn without replacement, and volume corrections were applied during data analysis.

Theoretical computation. The Vienna *ab initio* simulation package (VASP) was used to conduct the density functional theory (DFT) calculations. The generalized gradient approximation (GGA) of Perdew-Burke-Ernzerhof (PBE) exchange correlation functional was employed.[51–53] The DFT-D3 method was implemented to adjust the effect of nonbonding forces.[54] The cutoff energy was set to 400 eV, and the convergence criteria of energy and force were set to 10^{-5} eV and 0.03 eV $Å^{-1}$, respectively. The adsorption energy (E_a) of K_2S_2 and K_2S_4 on the different systems were calculated using the following equation:[55]

$$E_a = E_{tot} - E_{slab} - E_s \tag{9}$$

where E_{tot}, E_{slab}, E_s represent the energies of the substrate model adsorbed K_2S_2/K_2S_4, the clean slab, and K_2S_2/K_2S_4, respectively.

To investigate the interfacial behaviors of K ion and polysulfide species at the Ti_4O_7 surface, the *ab initio* molecular dynamics (AIMD) simulations were performed at room temperature. The time step was set to 1 fs, and the total simulation time was 10 ps. Furthermore, the climbing image nudge elastic band (CI-NEB) method was employed to calculate the diffusion energy barriers.[56]

Polysulfide adsorption test. UV–visible spectra were collected using a SEC2000 UV–visible Spectrophotometer. A 2.5 mM K_2S_2 or K_2S_4 solution was prepared by diluting with DI water in the glovebox. Equal molar amounts of KB or Ti_4O_7 were added to the solution under continuous stirring. After centrifugation, the supernatant was collected for UV–visible analysis. X-ray photoelectron spectroscopy (XPS) measurements were conducted on Thermo Scientific K-Alpha instrument. The prepared membranes were immersed in 1M K_2S_2 + 1M KOH solutions for 24 h. After being thoroughly washed with substantial DI water inside the glovebox, the membranes were dried in the glove oven before analysis.

Surface hydrophilicity. The contact angle was measured on a POWEREACH® JC2000D goniometer (Shanghai Zhongchen). 2 μL of DI water droplets were used for various membrane tests. ATR–FTIR was performed in the range of 500–4,000 cm^{-1}.

Zeta potential measurement. The zeta potentials of various membranes were measured using the SurPASS system (Anton Paar) via streaming potential measurements. Prior to measurement, the S@KB and POVE membranes were separately saturated in negolyte (1M K_2S_4 + 1M KOH) for 24 h in the glovebox. Subsequently, all the prepared membranes were rinsed with excessive DI water to eliminate free ions, followed by gently wiping the surface water.

S–Fe flow battery assembly and testing. Ni foam was cut into 3.4 × 3.5 cm^2 and boiled in 1 M K_2S_2 solution for several hours until the color of the Ni foam turned back evenly. The flow battery was assembled using a prepared membrane (5 × 5 cm^2), which was sandwiched between two PEEK frames. Pretreated graphite felt (500 °C for 10 h) was utilized as the positive electrode,[57] three pieces of pretreated nickel foam with an original thickness of 1.5 mm were stacked together and compressed to a total thickness of 3.0 mm as the negative electrode. A solution of 0.5 M $K_4[Fe(CN_6)]$ in 1 M KCl and a solution of 1 M K_2S_4 in 1 M KOH were used as the posolyte and negolyte, respectively, each with a volume of 10 mL. All the measurements were collected by LAND battery testing system with the voltage

window of 0.2–1.4 V. Self-discharge measurements were conducted by charging the flow battery to 50% state of charge (SOC) at 10 mA cm^{-2} and then resting at the open-circuit conditions. The rate performance test was conducted with current densities ranging from 20 mA cm^{-2} to 200 mA cm^{-2}. Long-term cycling performance was tested under 40 mA cm^{-2}. The output power density was measured under 100% SOC. Specifically, the flow battery was charged at 40 mA cm^{-2}, then discharged for 60 s at specified current densities (10–420 mA cm^{-2}), during which the battery voltage was continuously recorded.

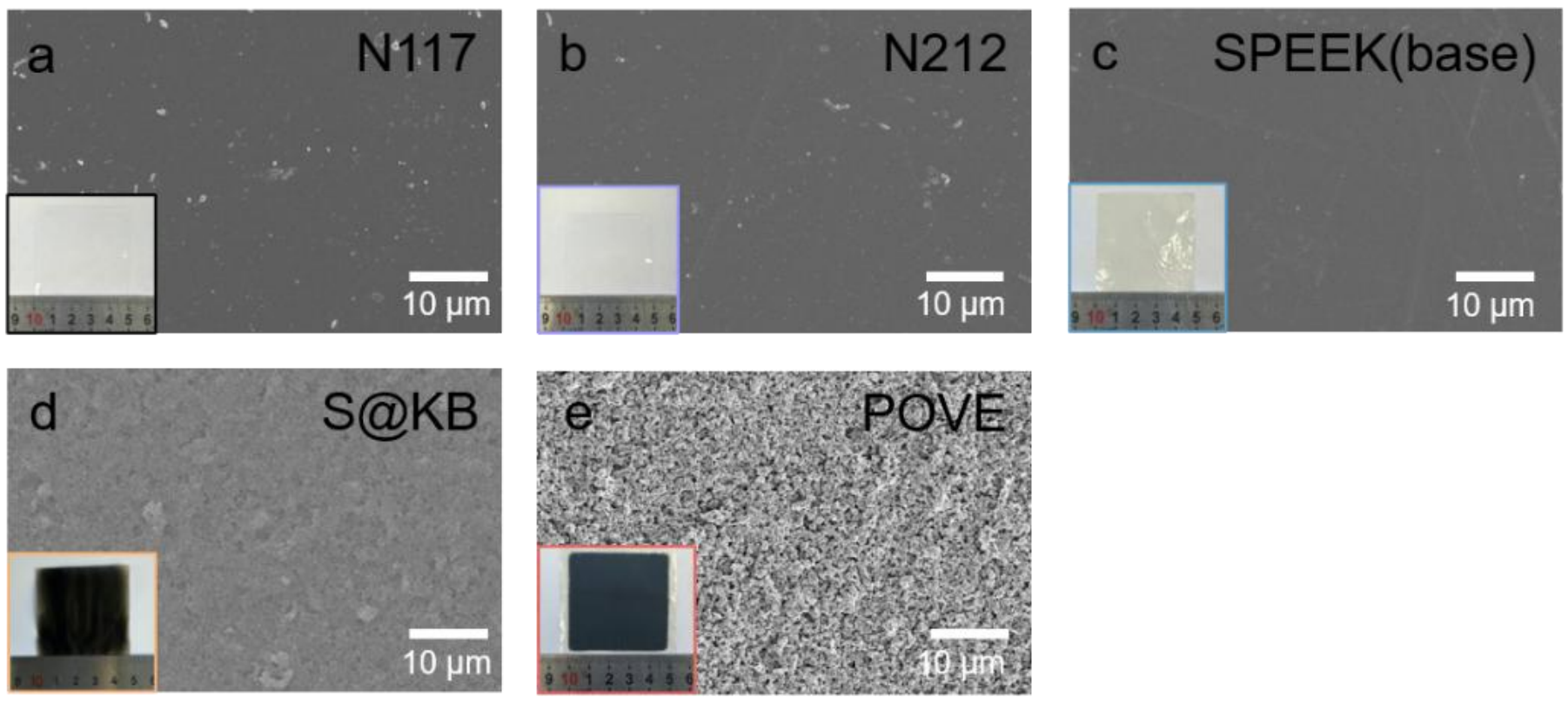

Figure S1. Scanning electron microscopy (SEM) images and digital photographs of various ion-exchange membranes (IEMs). a) Nafion 117 (N117) membrane; b) Nafion 212 (N212) membrane; c) Sulfonated poly (ether ether ketone) (SPEEK) membrane; d) Carbon-based ketjen black (KB)-coated SPEEK (S@KB) membrane; e) Ti_4O_7-coated SPEEK (POVE) membranes.

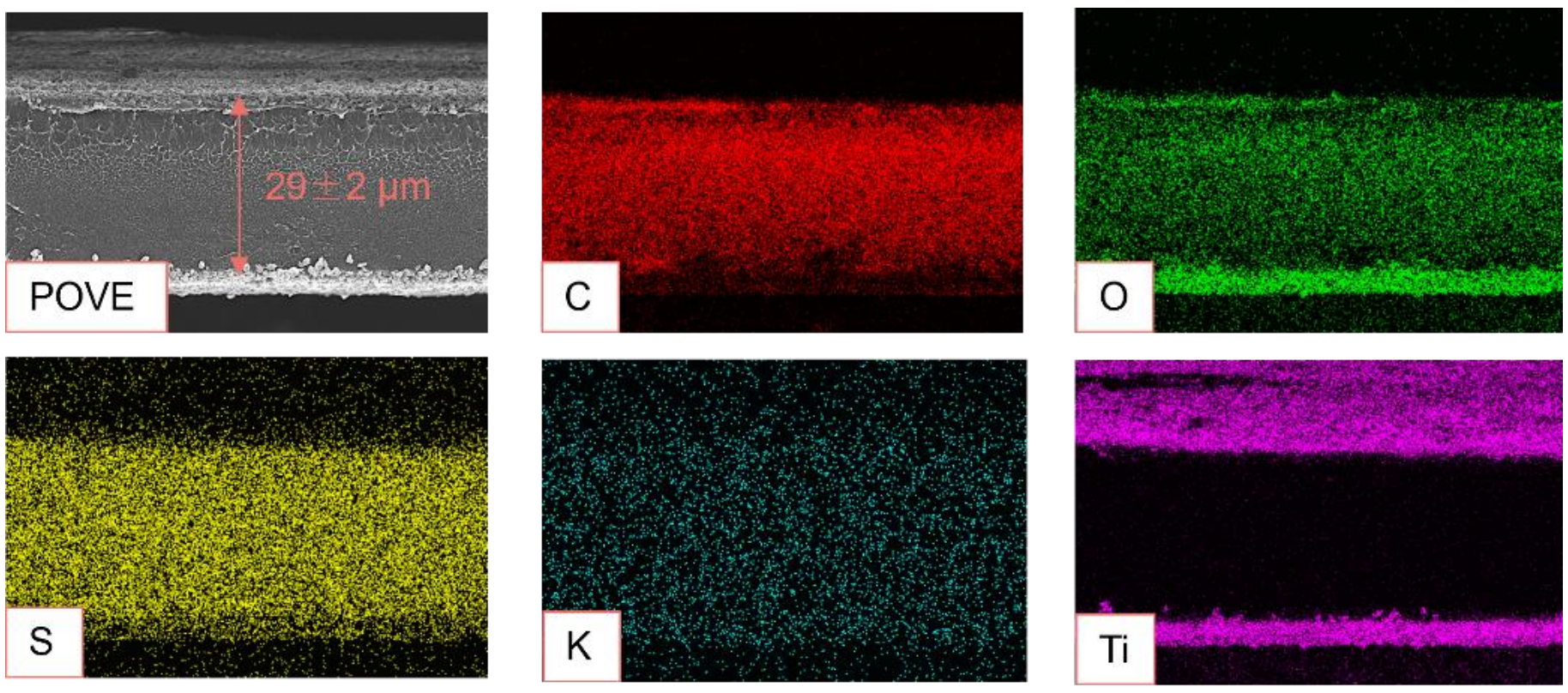

Figure S2. Energy dispersive X-ray spectroscopy (EDS) images of sandwich-structured POVE membrane.

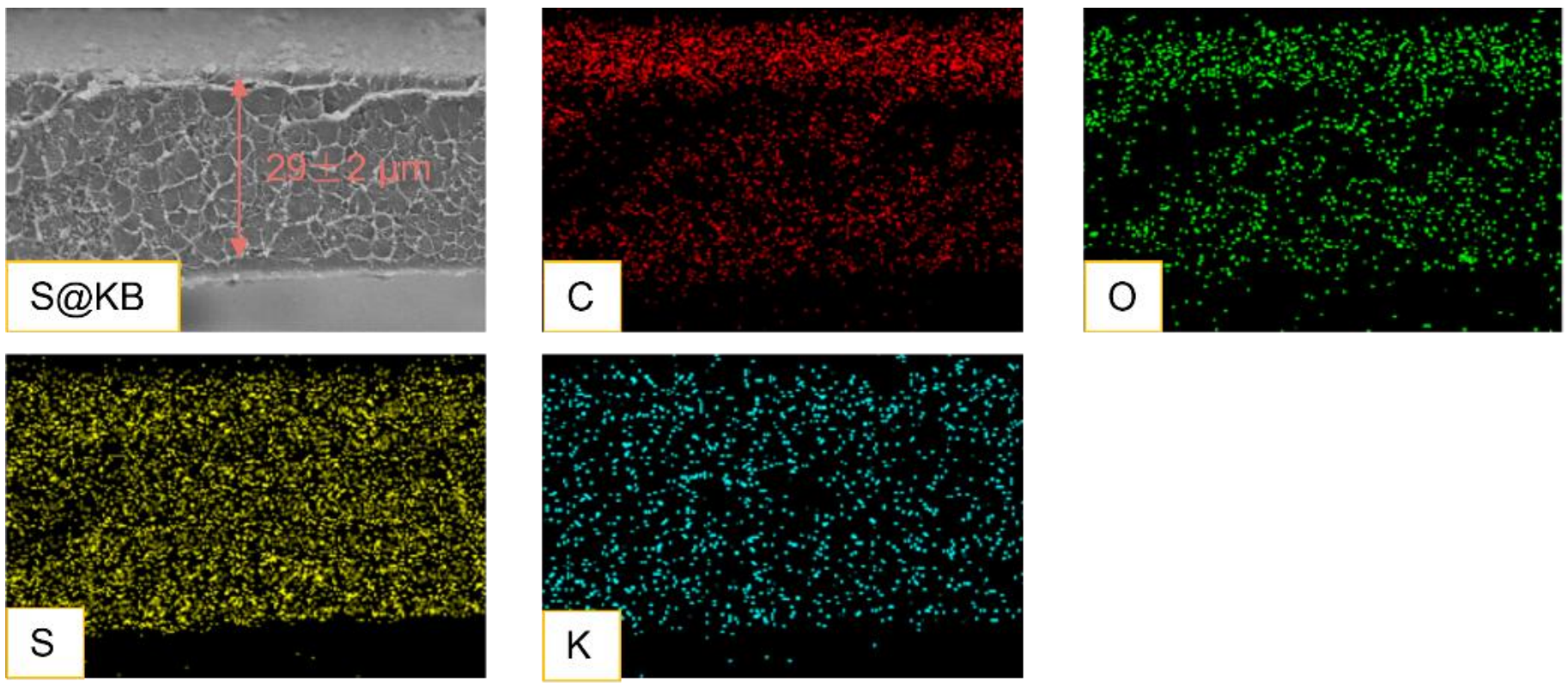

Figure S3. EDS images of sandwich-structured S@KB membrane.

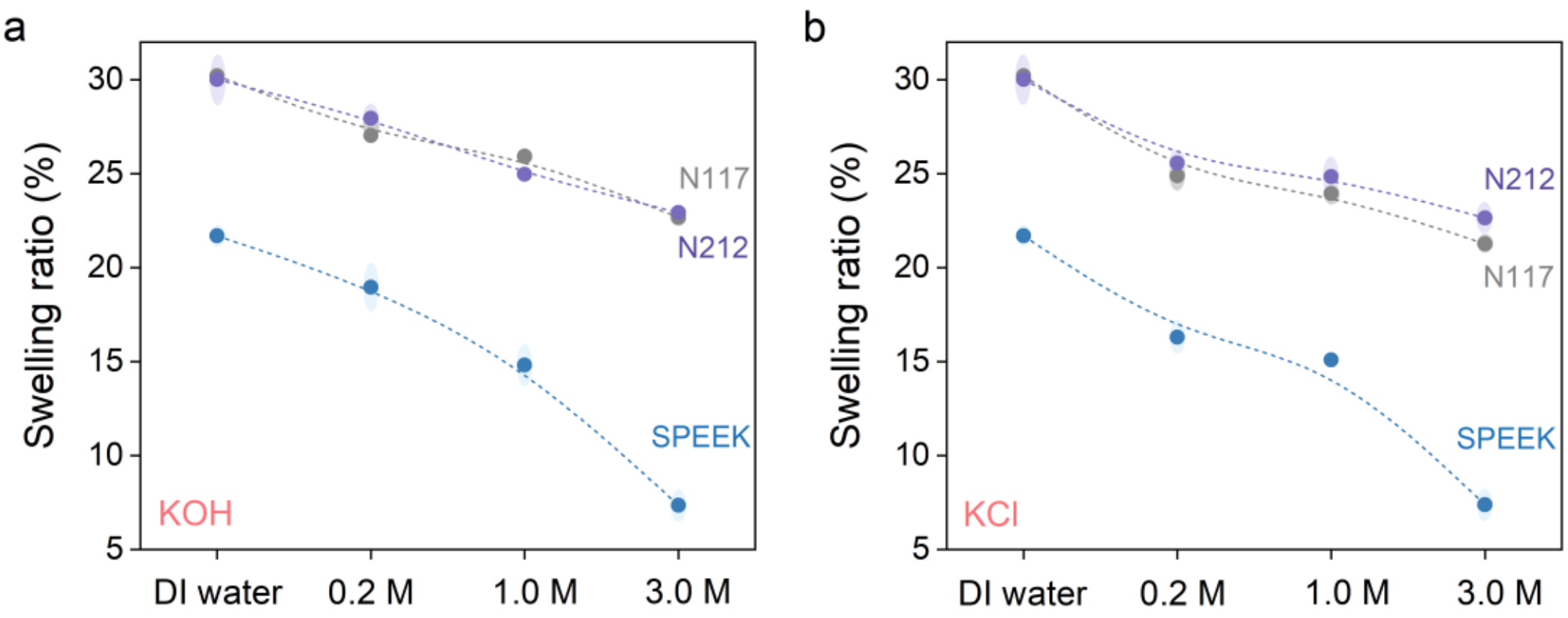

Figure S4. Concentration-dependent swelling ratios of N117, N212, and SPEEK membranes in KOH and KCl solutions, respectively.

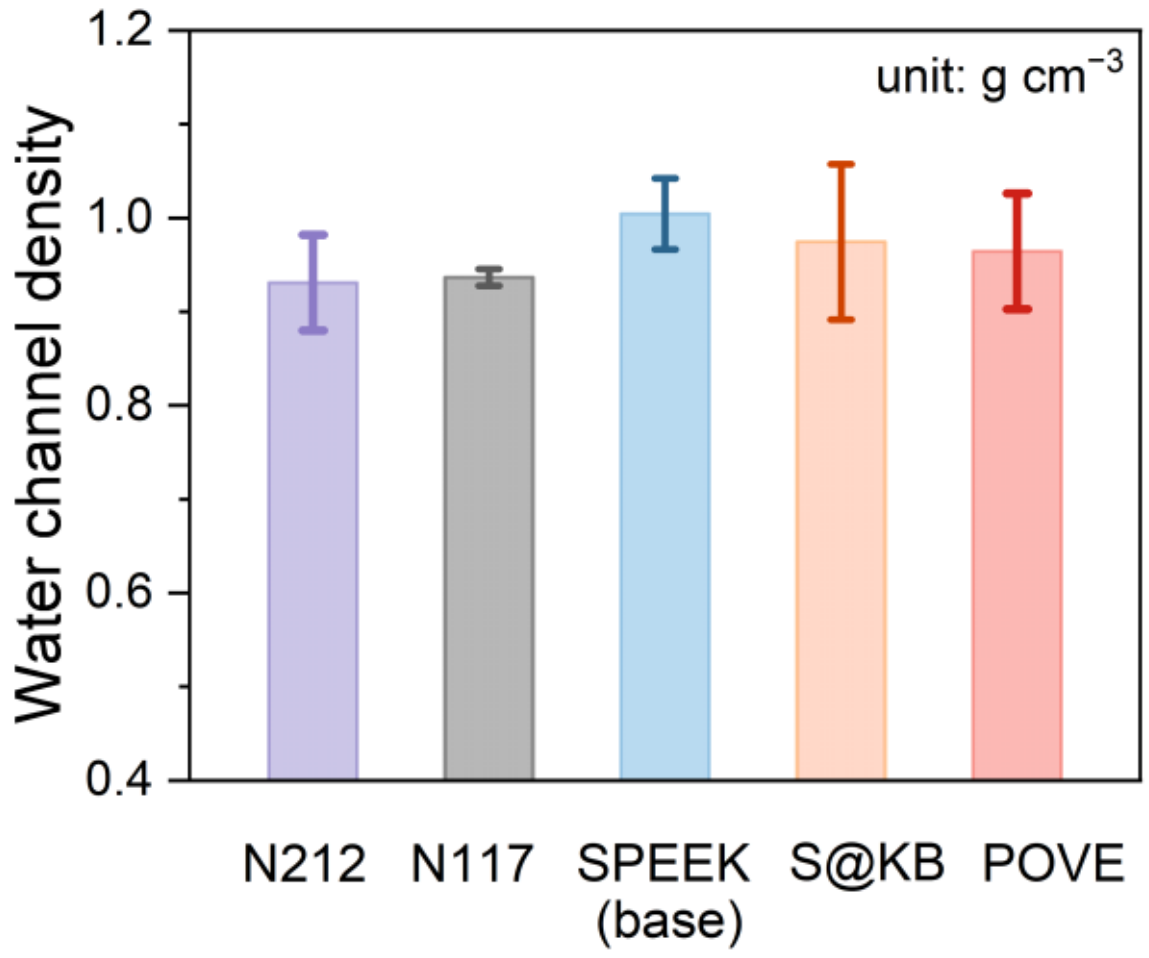

Figure S5. Water channel density of various IEMs.

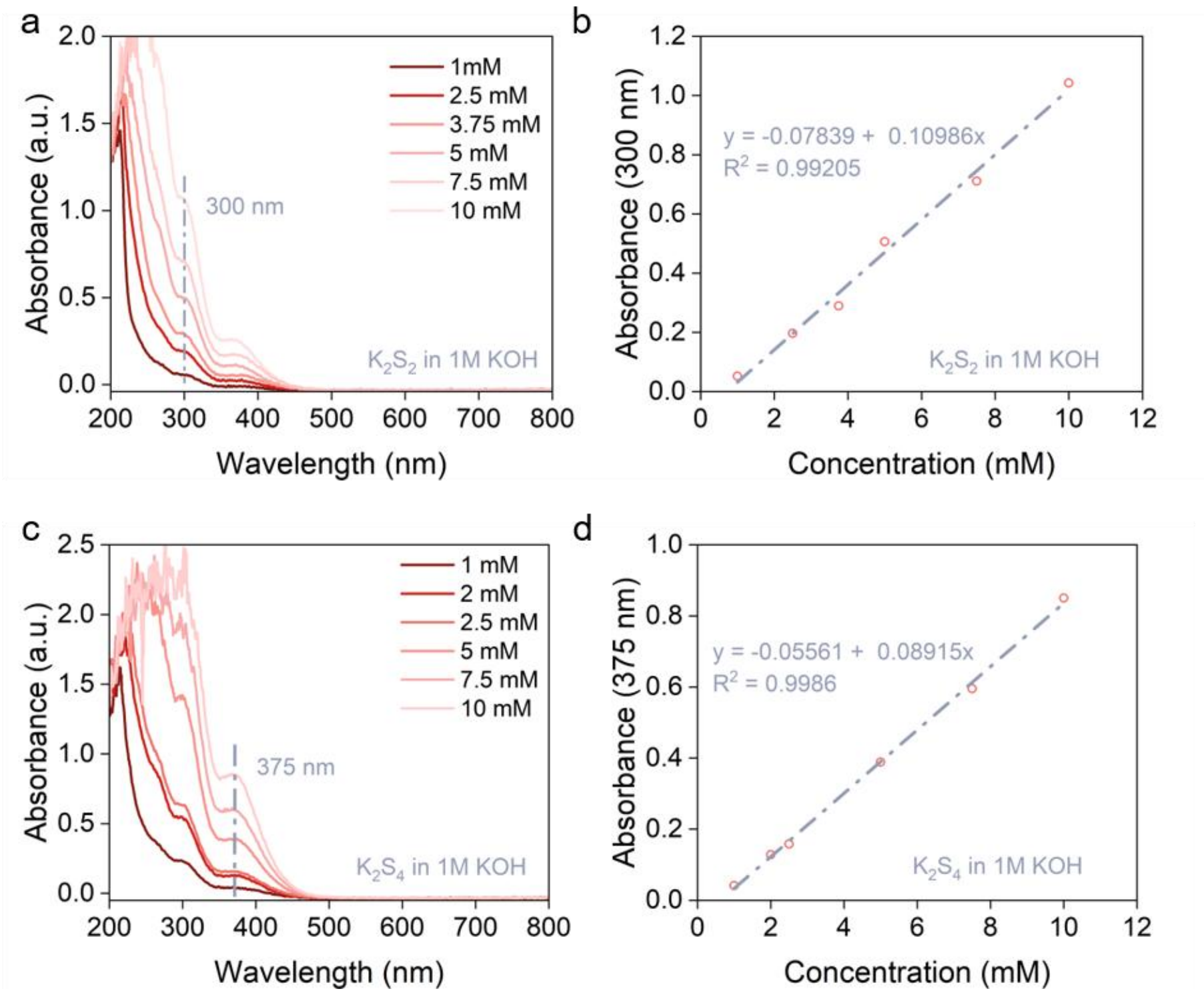

Figure S6. Standard UV–visible absorption spectra of polysulfide ions (S_2^{2-}/S_4^{2-}) in 1M KOH solutions. a) S_2^{2-} with different concentrations; b) Linear fitting curve of S_2^{2-}; c) S_4^{2-} with different concentrations; d) Linear fitting curve of S_4^{2-}.

182 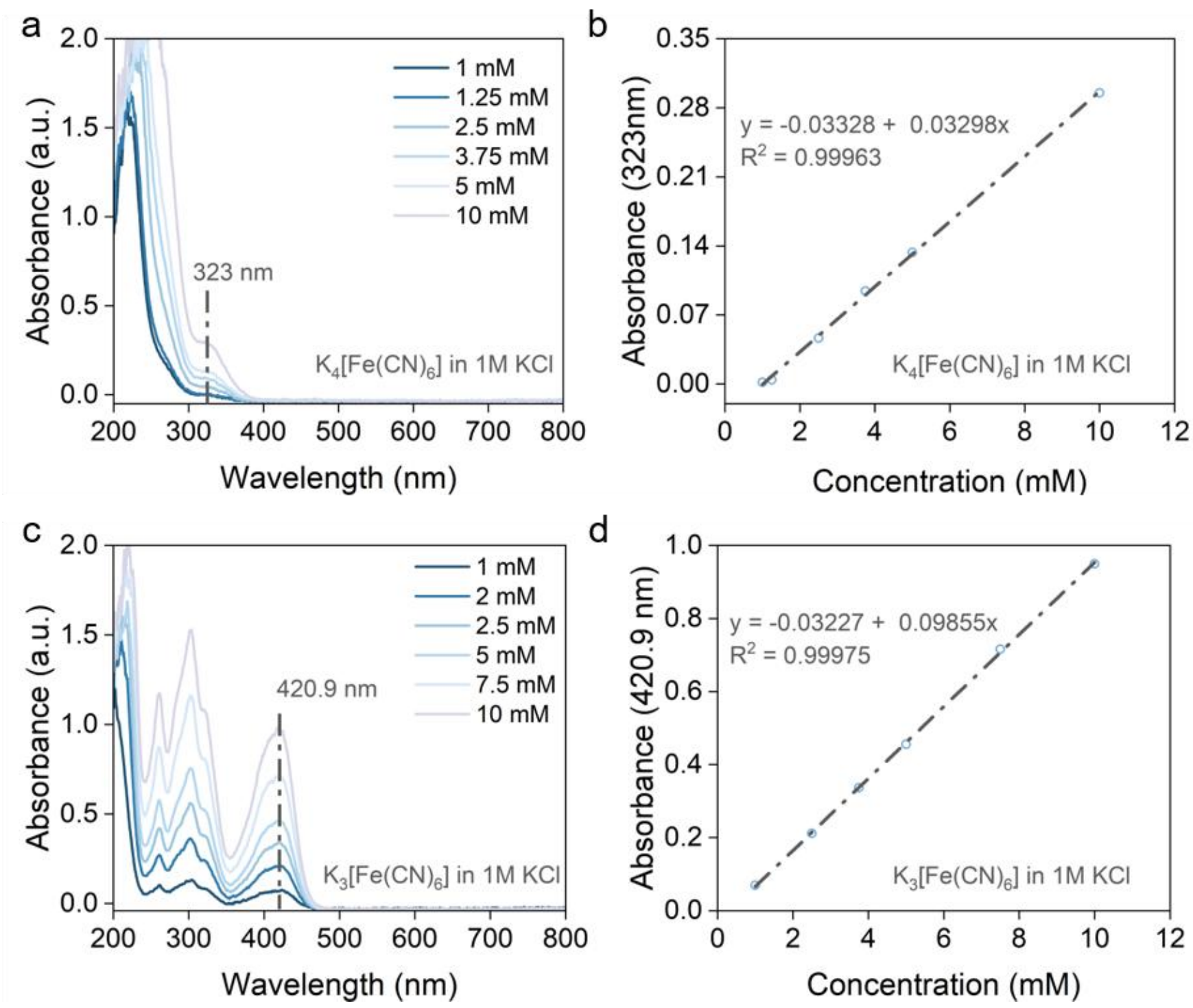

183 Figure S7. Standard UV–visible absorption spectra of ferro-/ferricyanide ions ($[Fe(CN)_6]^{4-}$/$[Fe(CN)_6]^{3-}$) in 1M KCl solutions. a) $[Fe(CN)_6]^{4-}$ with different concentrations; b) Linear fitting curve of $[Fe(CN)_6]^{4-}$; c) $[Fe(CN)_6]^{3-}$ with different concentrations; d) Linear fitting curve of $[Fe(CN)_6]^{3-}$.

186

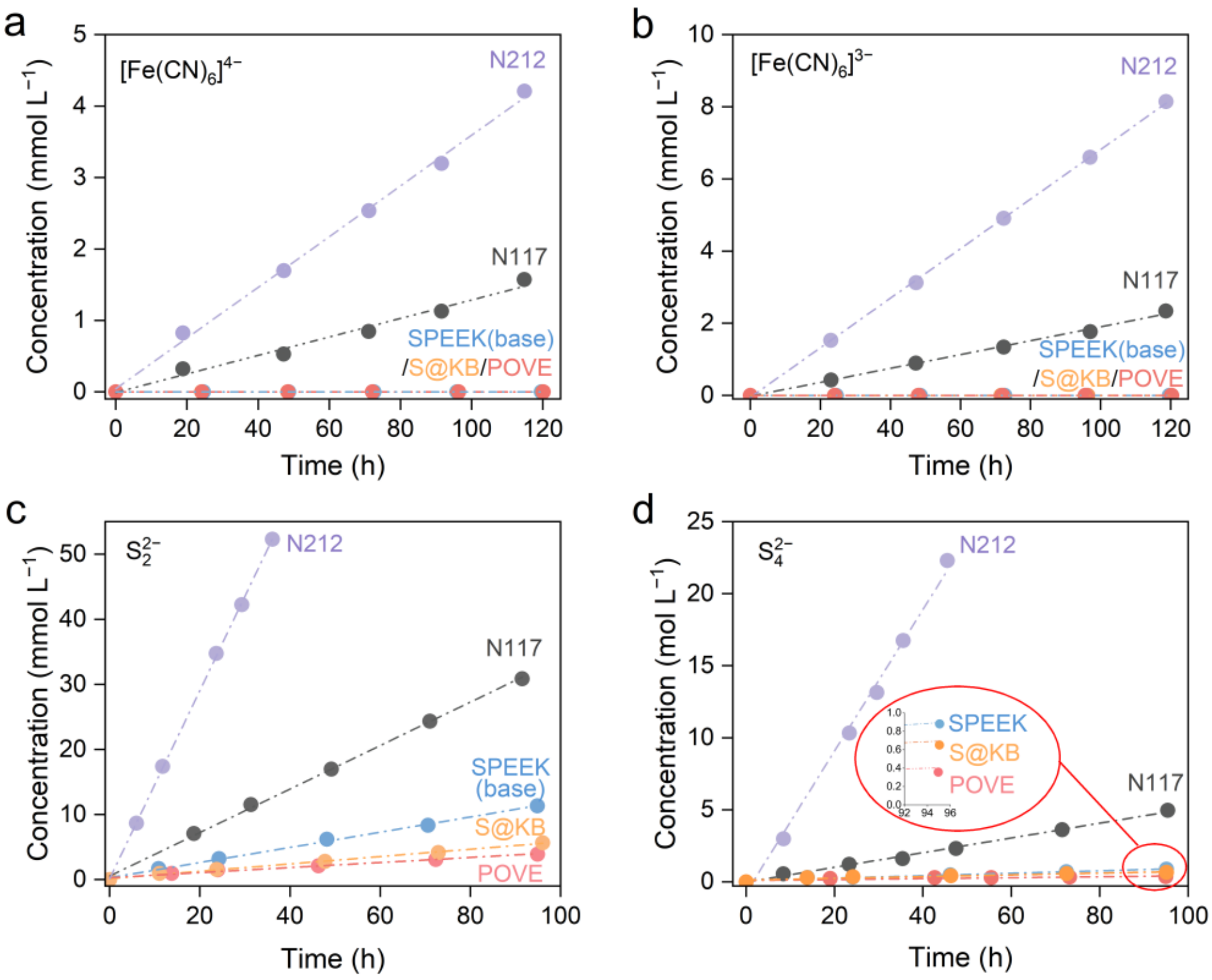

Figure S8. Time-dependent permeation profiles of various IEMs. a) $[Fe(CN)_6]^{4-}$; b) $[Fe(CN)_6]^{3-}$; c) S_2^{2-}; d) S_4^{2-}.

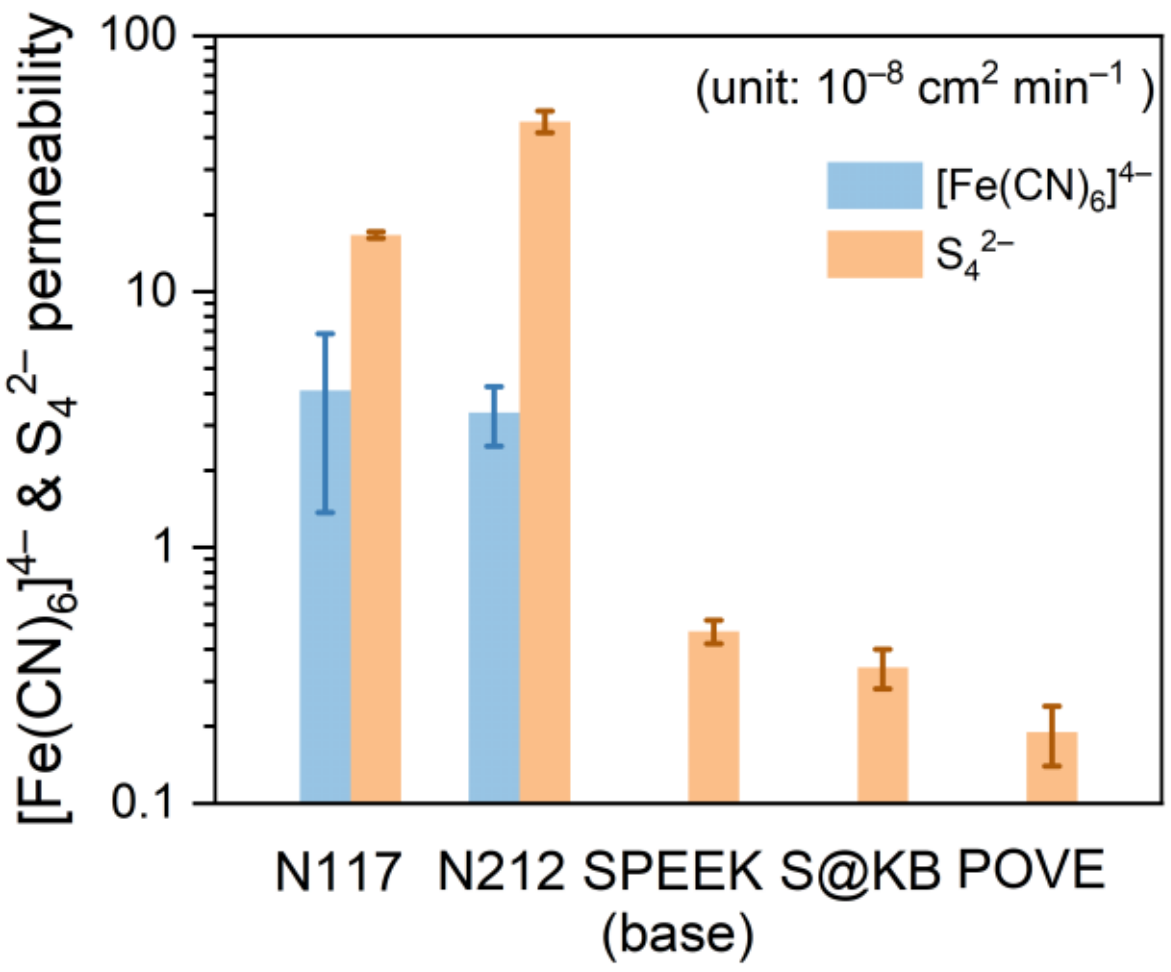

Figure S9. Ionic permeability of various IEMs.

(Notes: The absorbance spectra of $[Fe(CN)_6]^{4-}$ in hydrocarbon-based SPEEK membranes were below the detection limit of the UV–visible spectrophotometer during the testing period.)

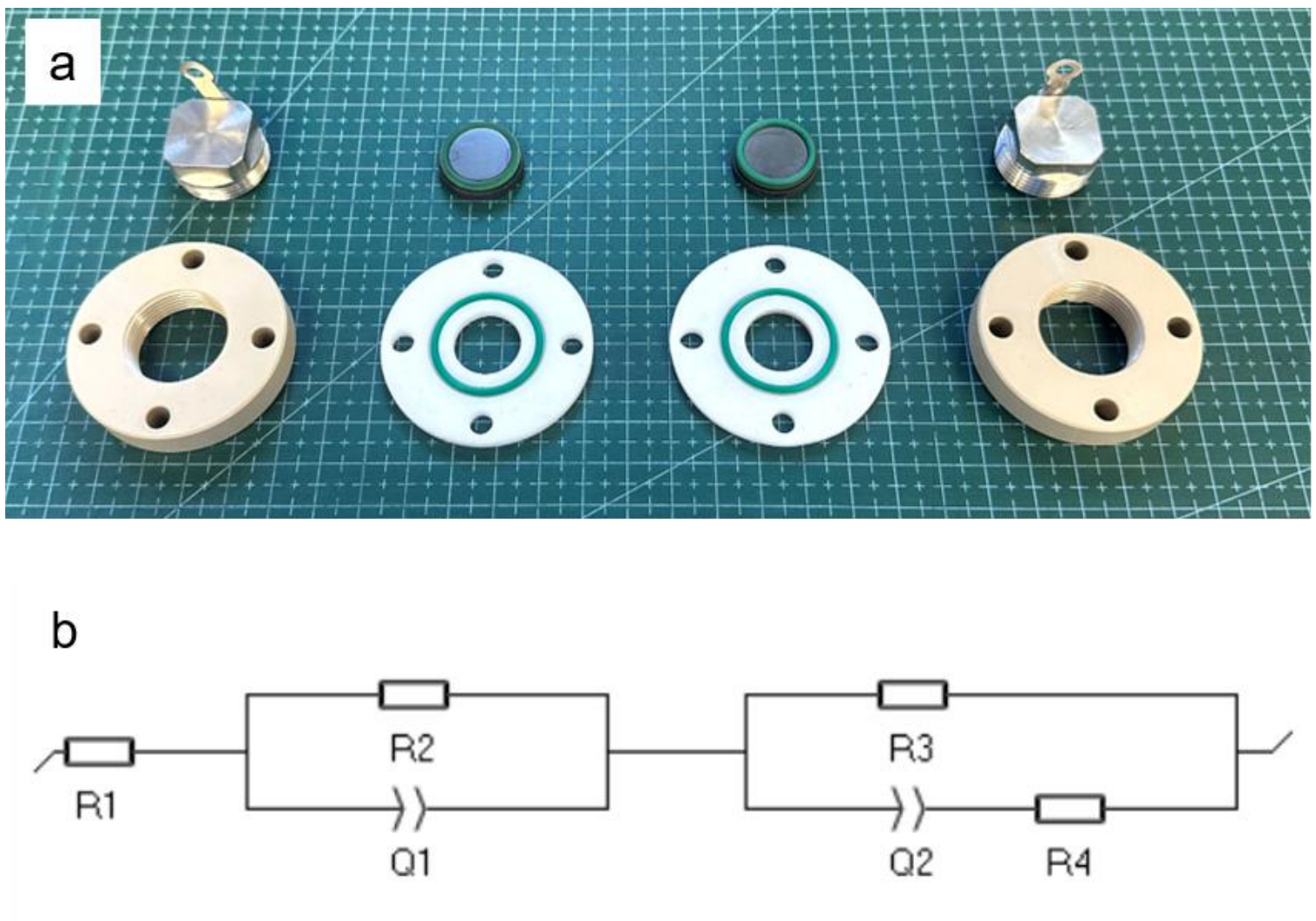

Figure S10. Electrochemical impedance spectroscopy (EIS) testing for ionic conductivity and area-specific resistance (ASR). a) Digital images of cell components; b) Equivalent circuit model for fitting the Nyquist plots.[58]

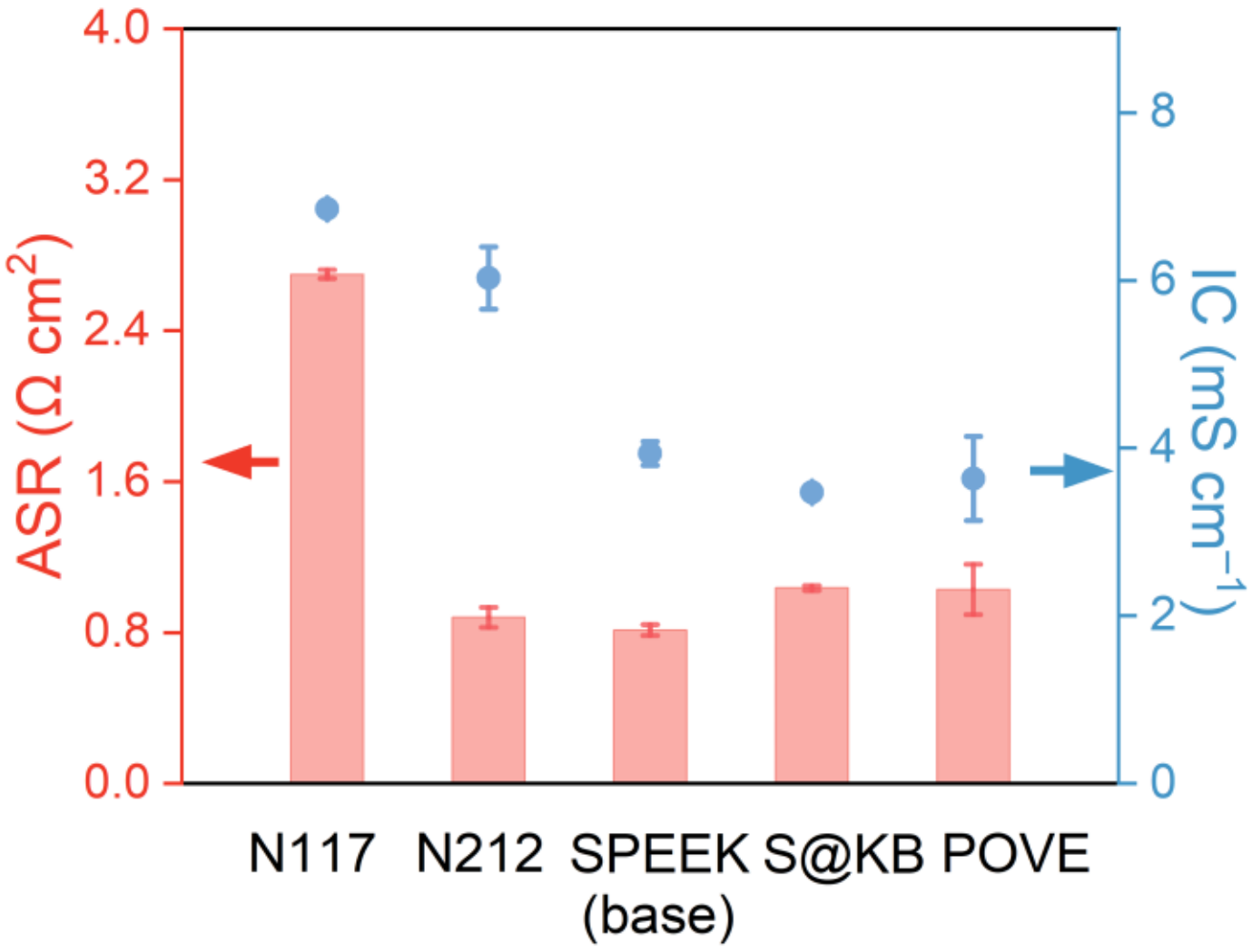

Figure S11. Area-specific resistance (ASR) and ionic conductivity (IC) of various IEMs in 1M KCl solution.

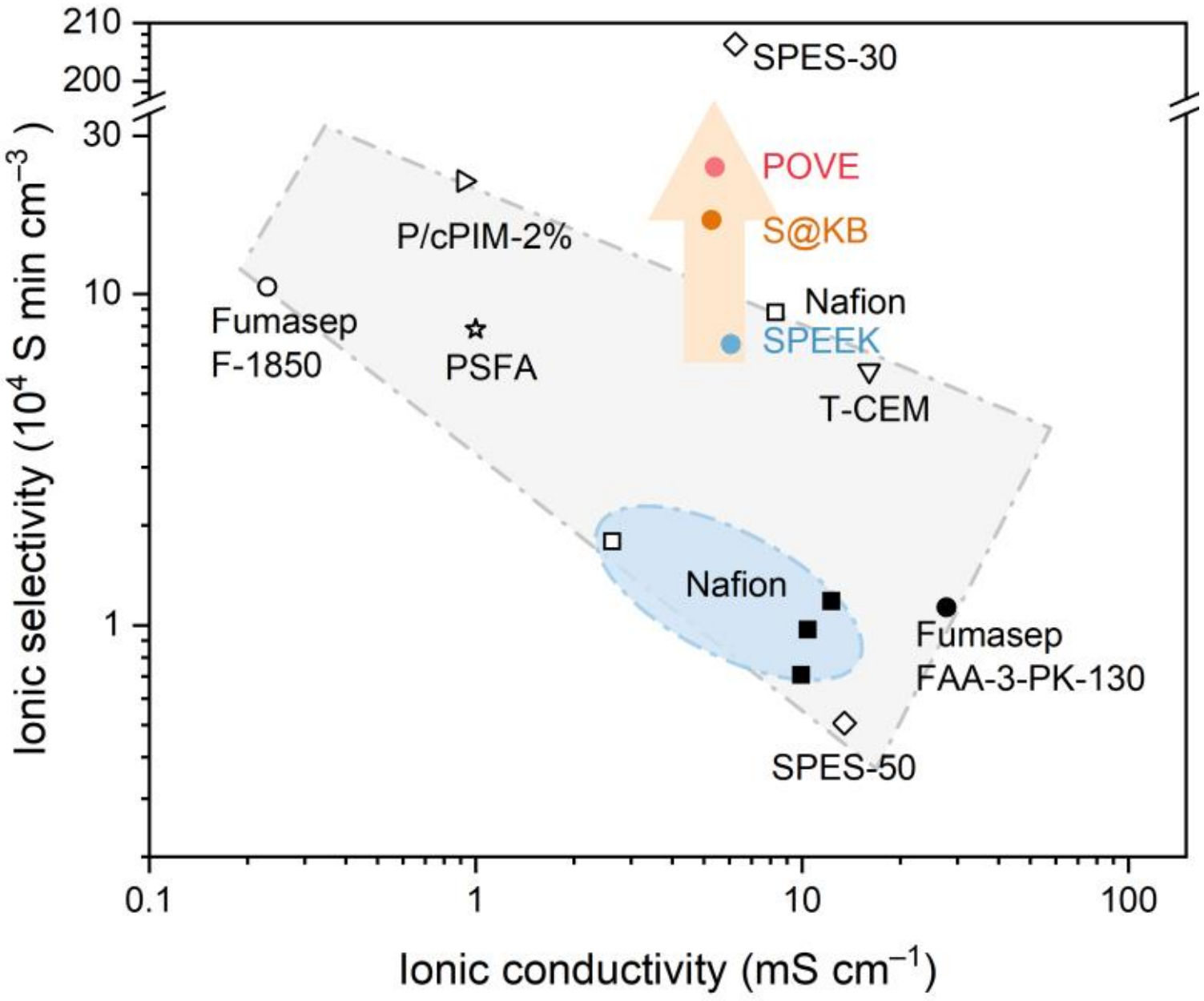

Figure S12. Ionic conductivity and selectivity of various recently reported membranes. (Detailed data are provided in Table S3. Solid symbols indicate measurements performed under an argon-protected atmosphere, whereas hollow symbols indicate measurements performed under ambient air or without a specified atmosphere-protection condition.)

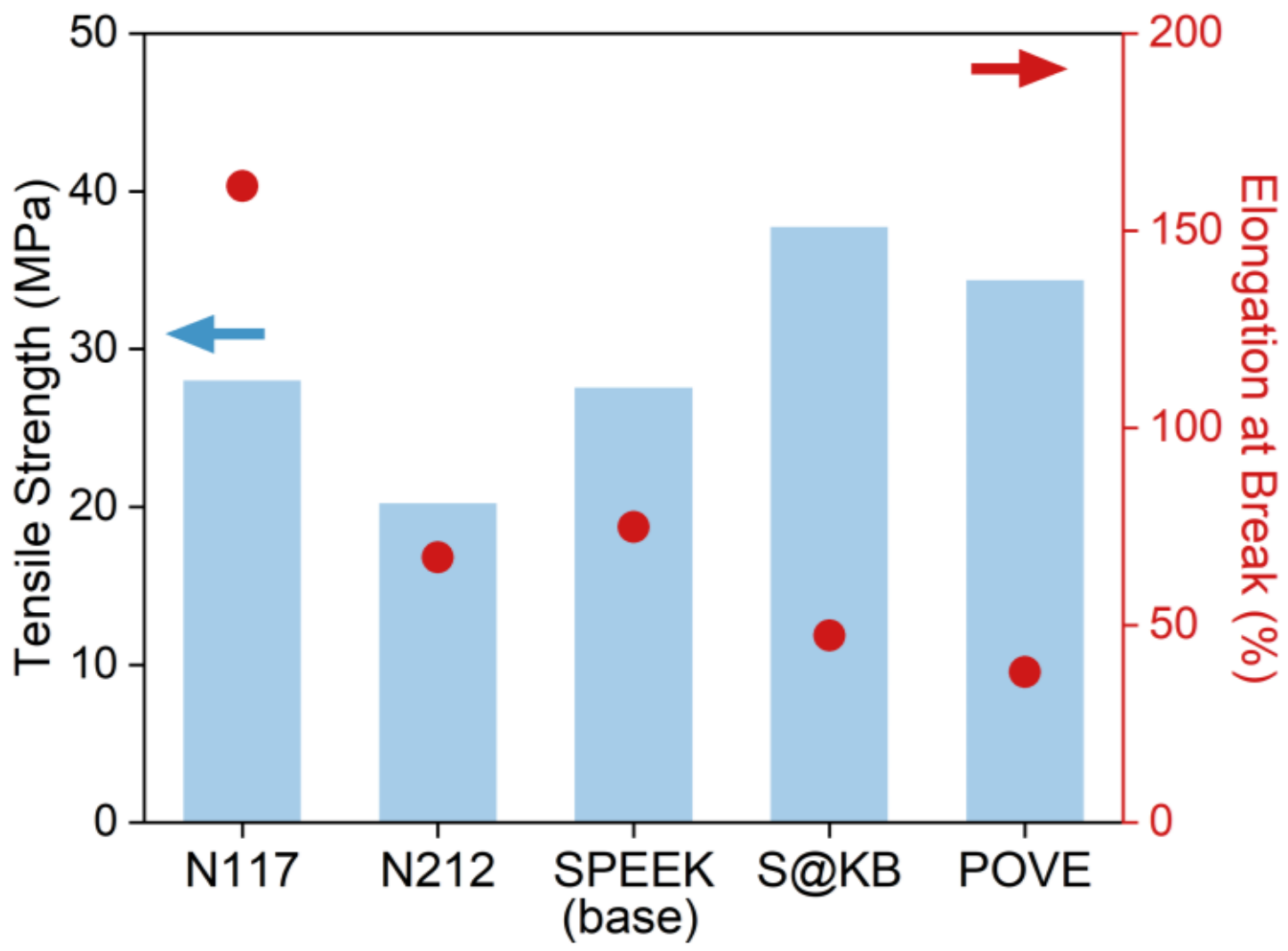

Figure S13. Tensile strength and elongation at break properties of various IEMs.

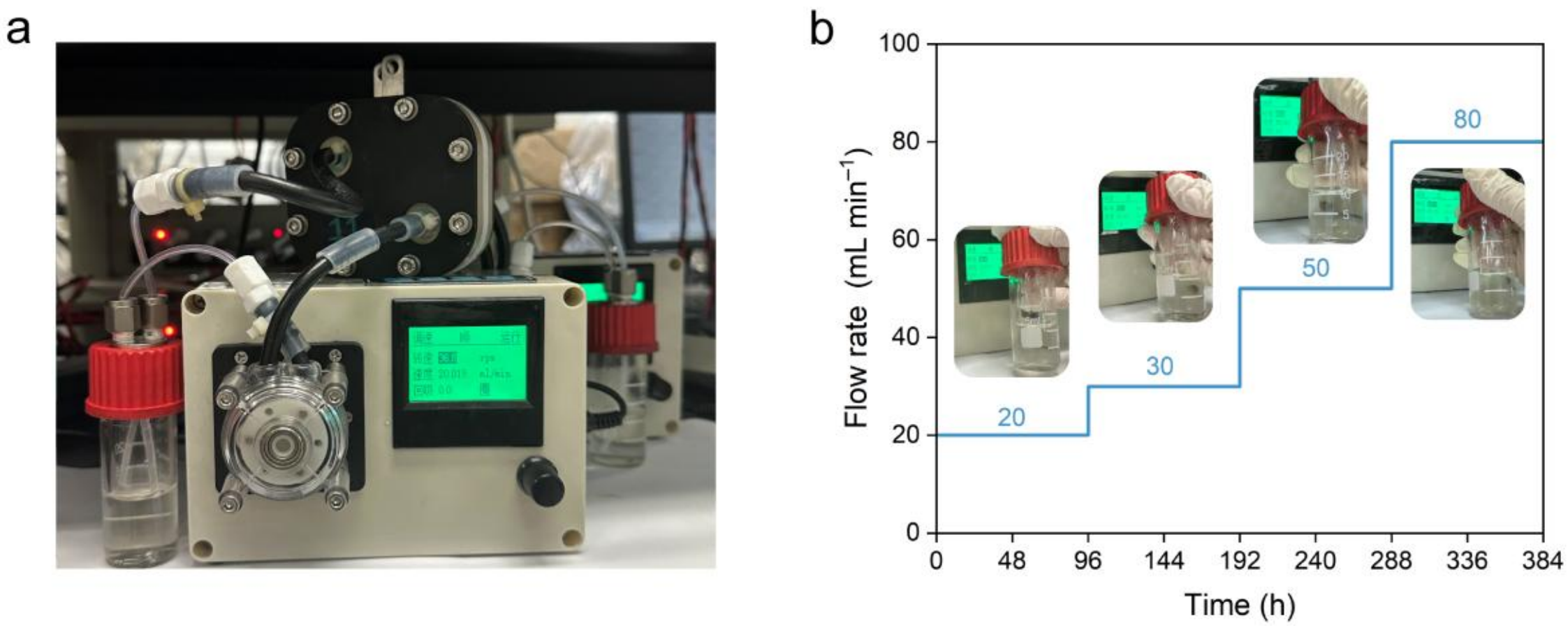

Figure S14. Mechanical stability of POVE membrane. a) Digital image of the water flushing experiment of the flow battery configuration; b) Digital images of the water reservoir after flushing under different flow rates.

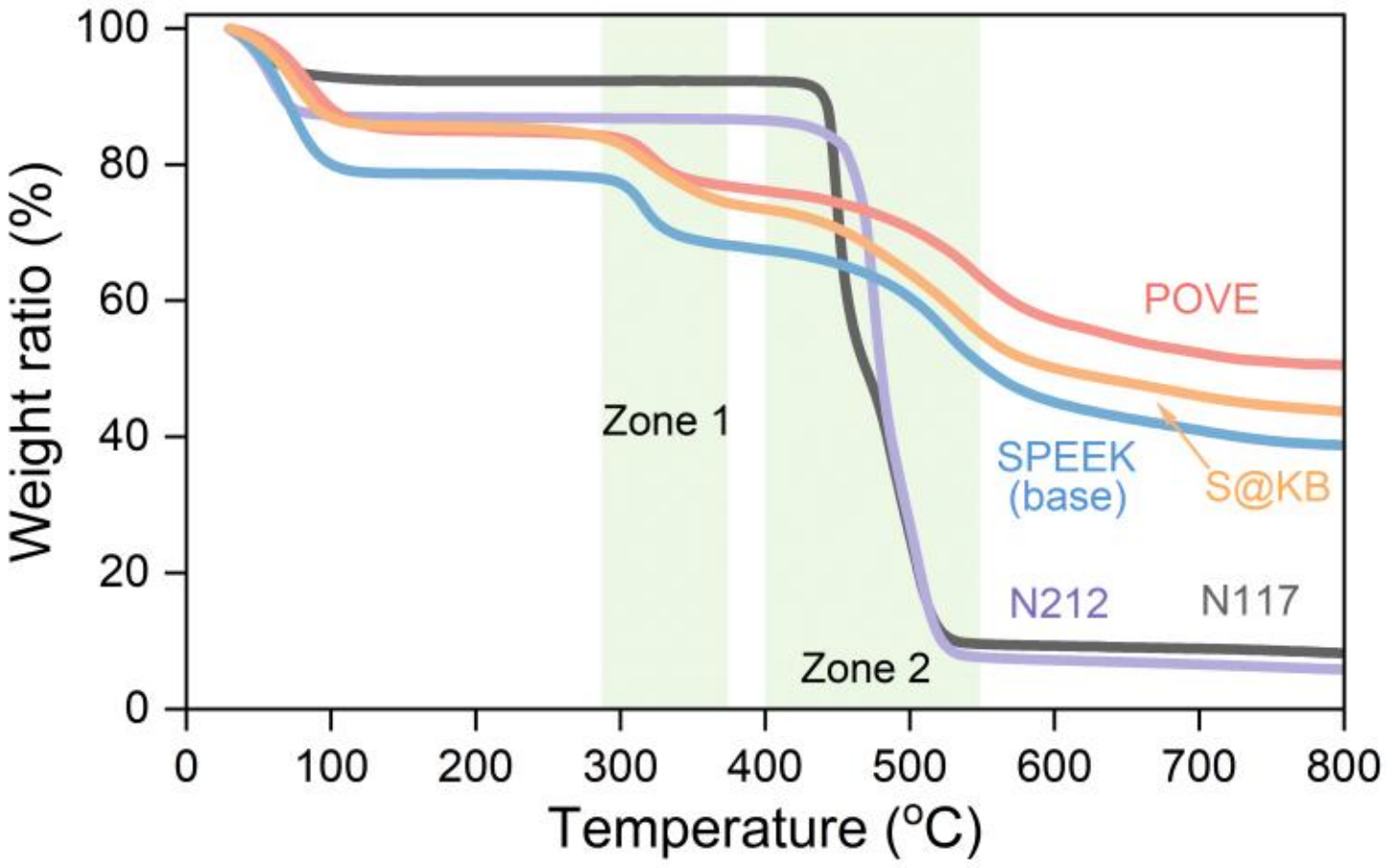

Figure S15. Thermal stability of various IEMs.

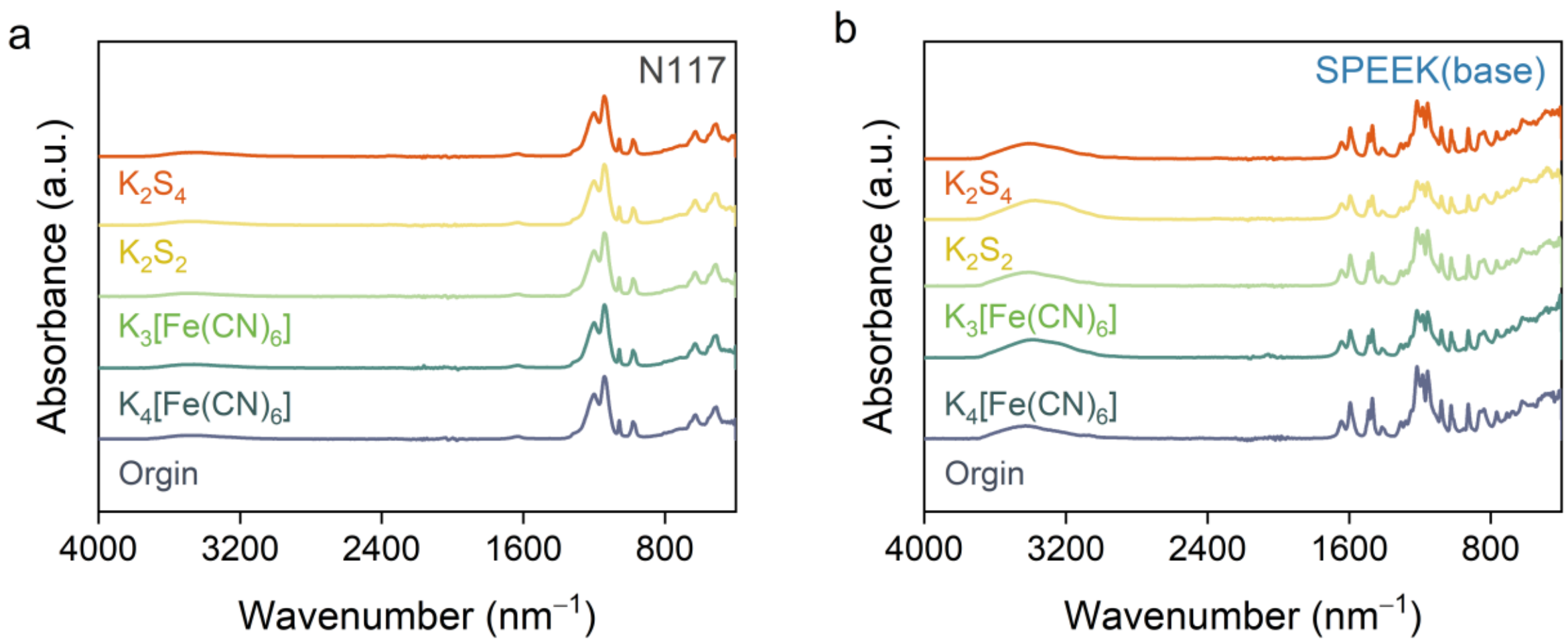

Figure S16. Attenuated total reflectance Fourier–transform infrared (ATR–FTIR) spectra of various membranes. a) N117 before and after immersion in different electrolyte solutions for 60 days; b) SPEEK (base) membrane before and after immersion in different electrolyte solutions for 60 days.

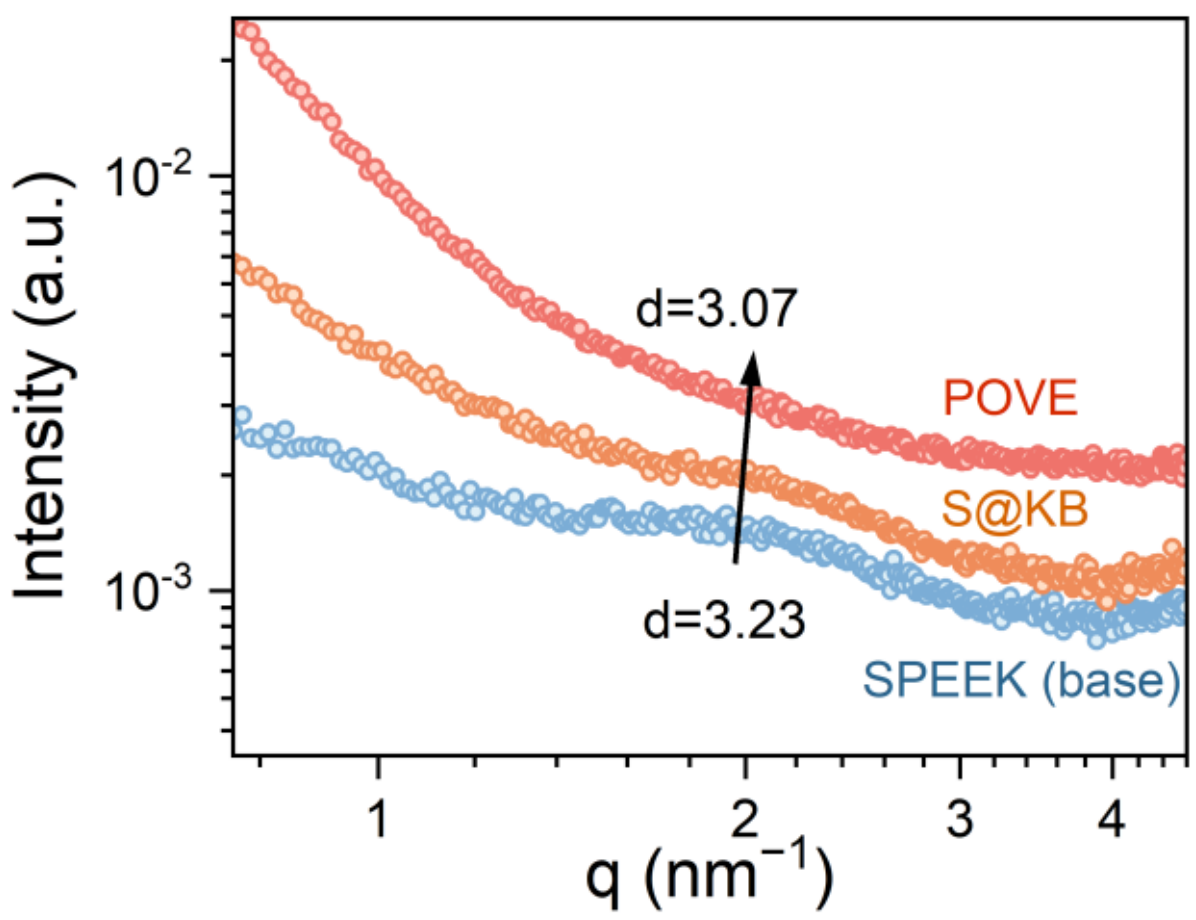

Figure S17. Small-angle X-ray scattering (SAXS) profiles of various SPEEK-based membranes.

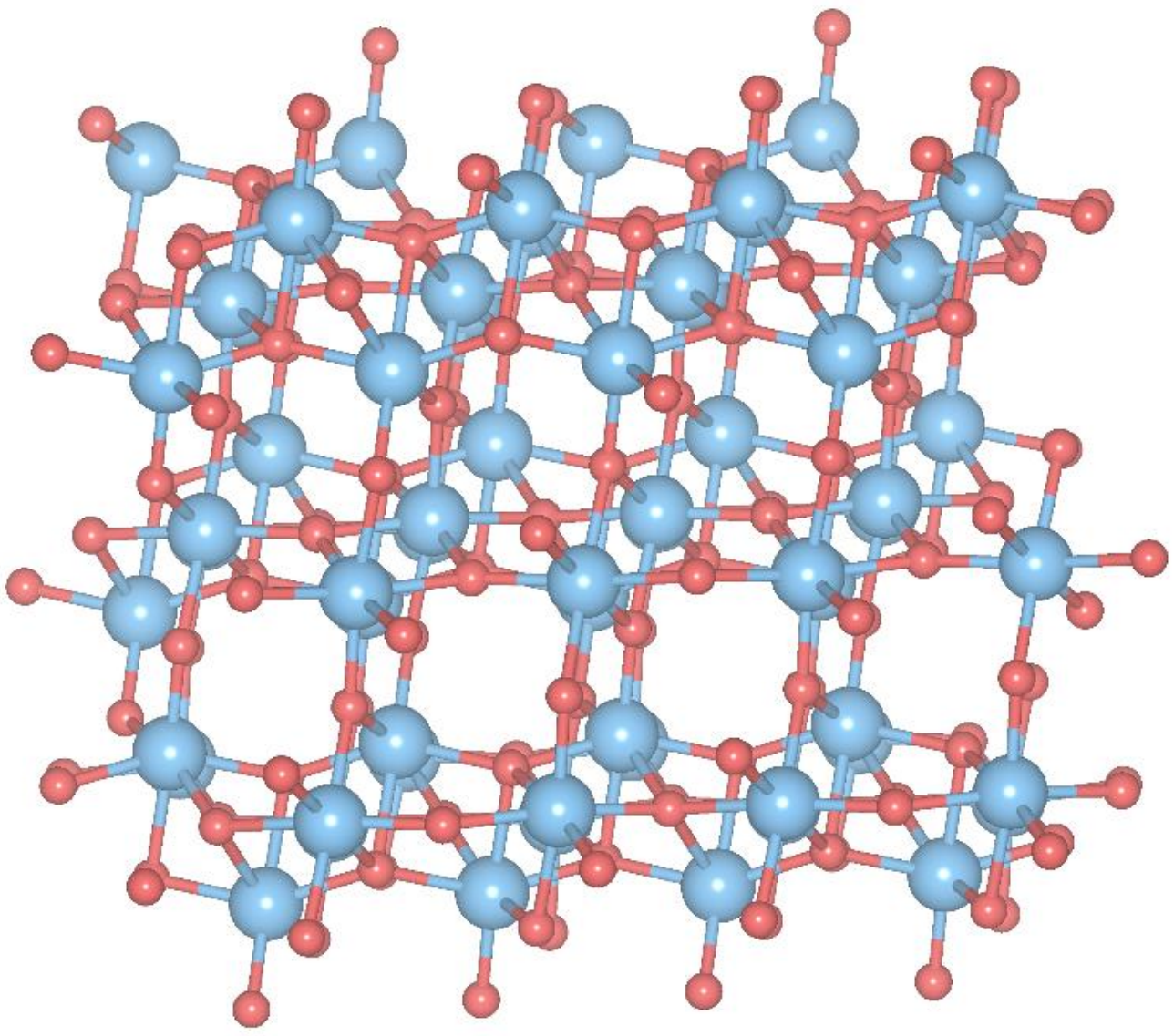

Figure S18. The structure of Ti_4O_7 (red: O, blue: Ti).

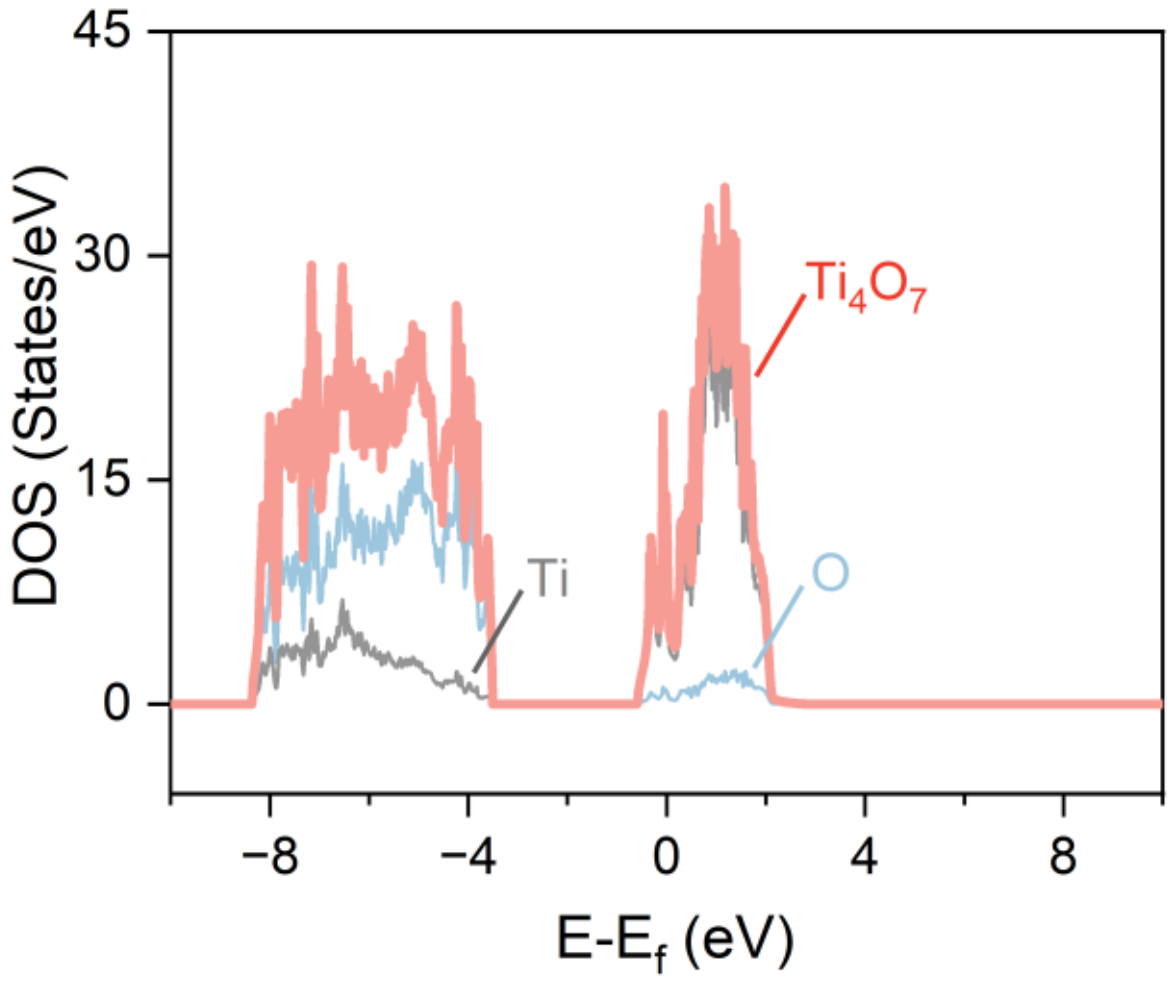

Figure S19. The density of states (DOS) of Ti_4O_7 materials.

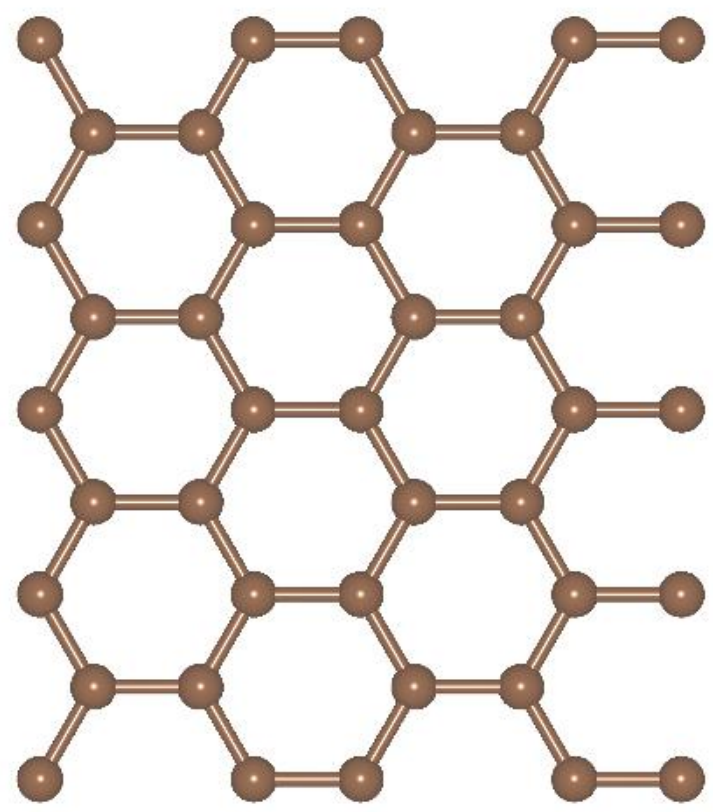

Figure S20. The structure of carbon-based KB materials.

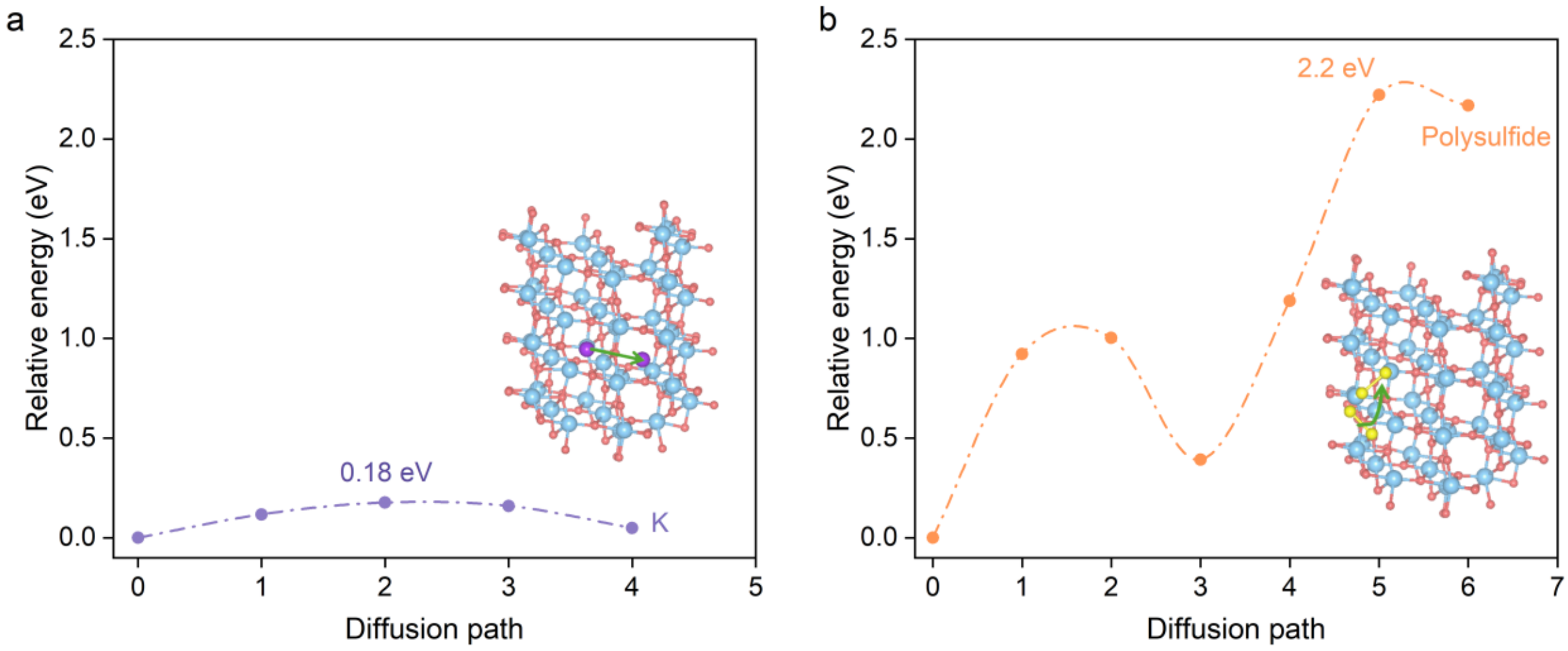

Figure S21. The schematic diagram the diffusion pathway and the corresponding energy barrier profiles of a) K and b) polysulfide diffusion paths on Ti_4O_7 surface.

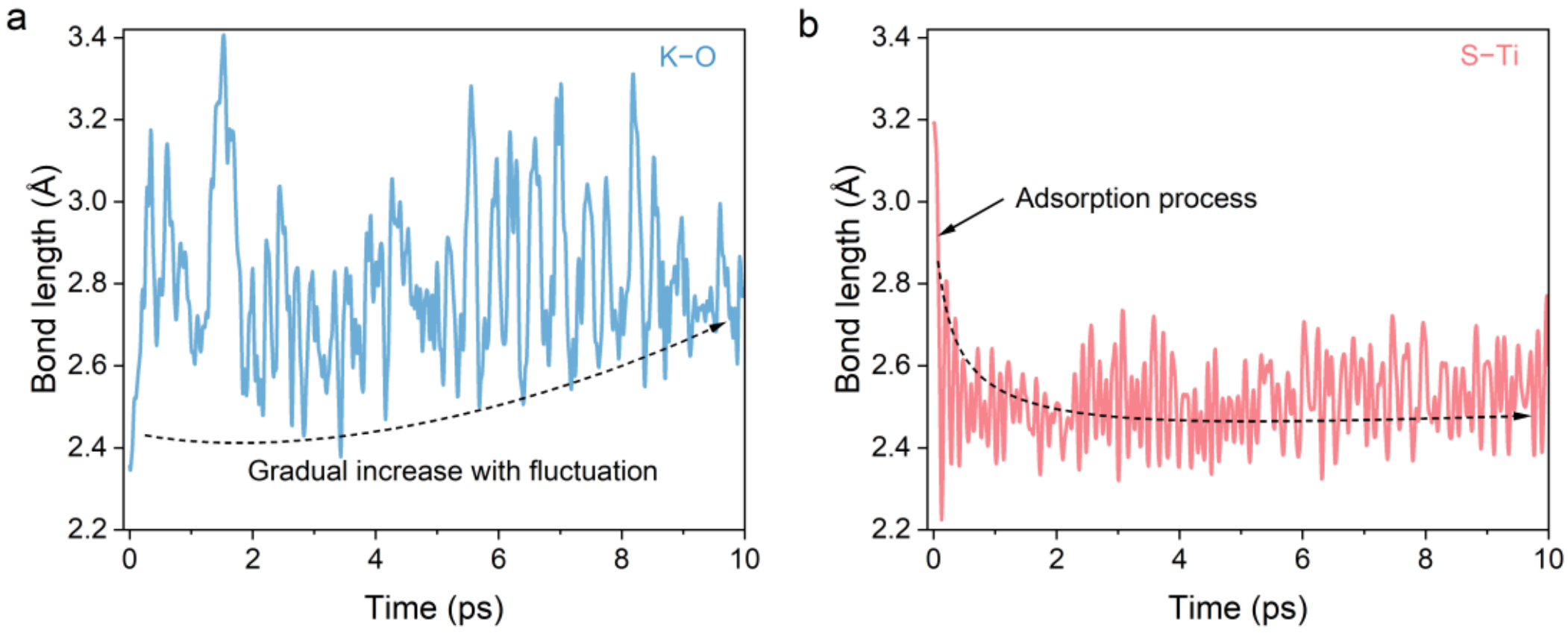

Figure S22. Dynamic interfacial bond length of K and S atoms near the Ti_4O_7 surface during AIMD simulations (Dash lines indicate the corresponding trends).

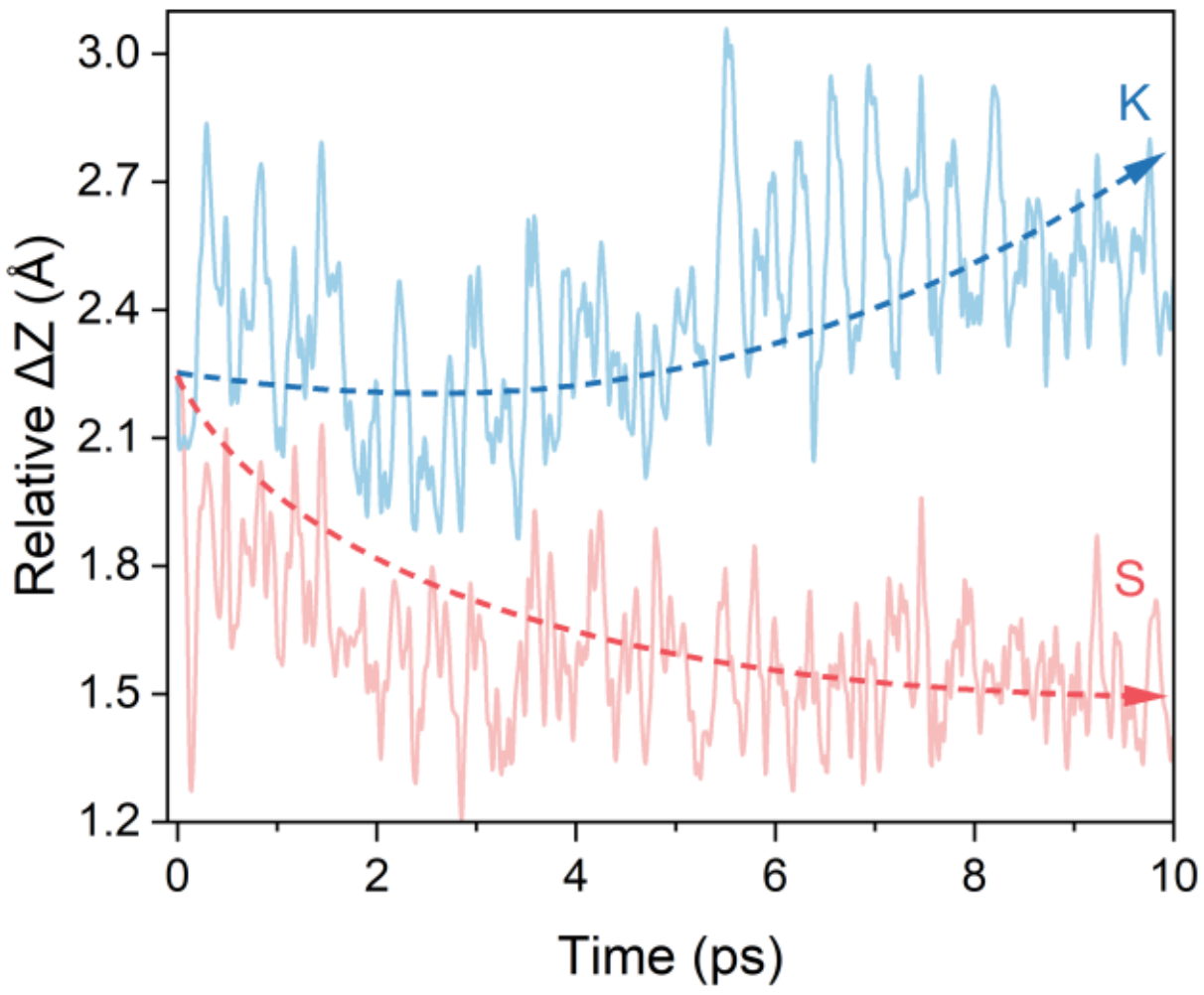

Figure S23. Dynamic ΔZ (vertical distance) of K and S atoms near the Ti_4O_7 surface during AIMD simulations (Dash lines indicate the corresponding trends).

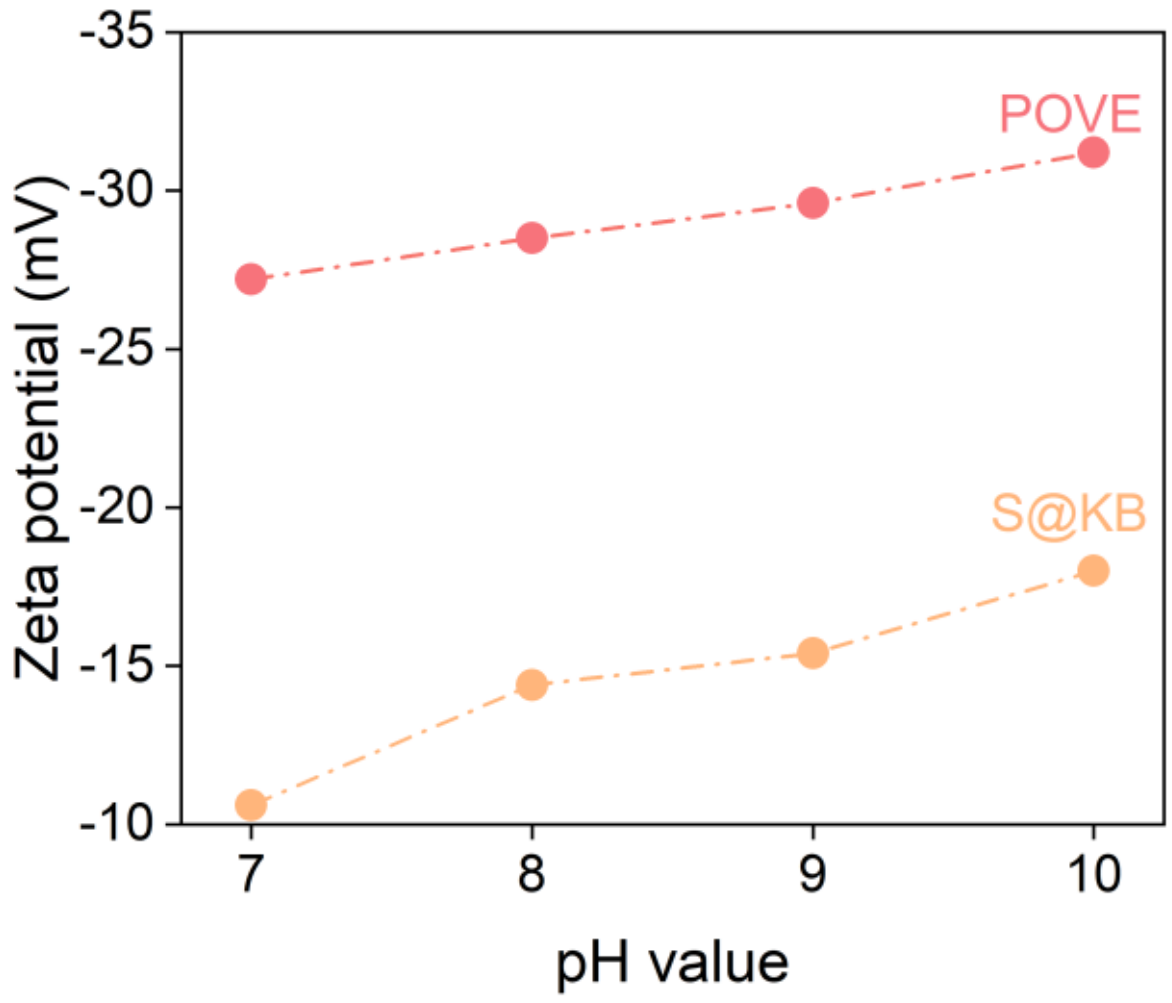

Figure S24. The zeta potentials of S@KB and POVE membranes.

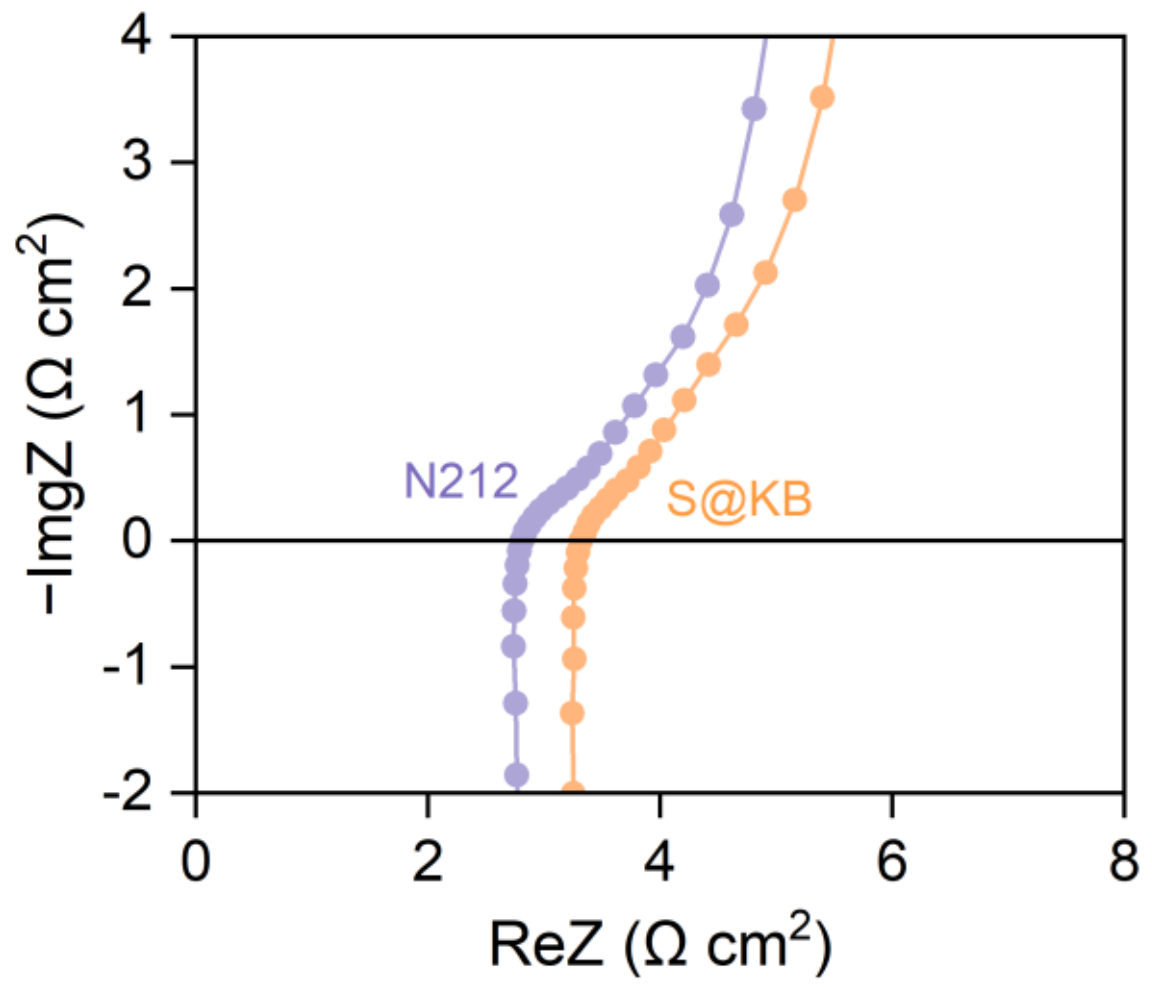

Figure S25. Nyquist plots of various IEMs by EIS test.

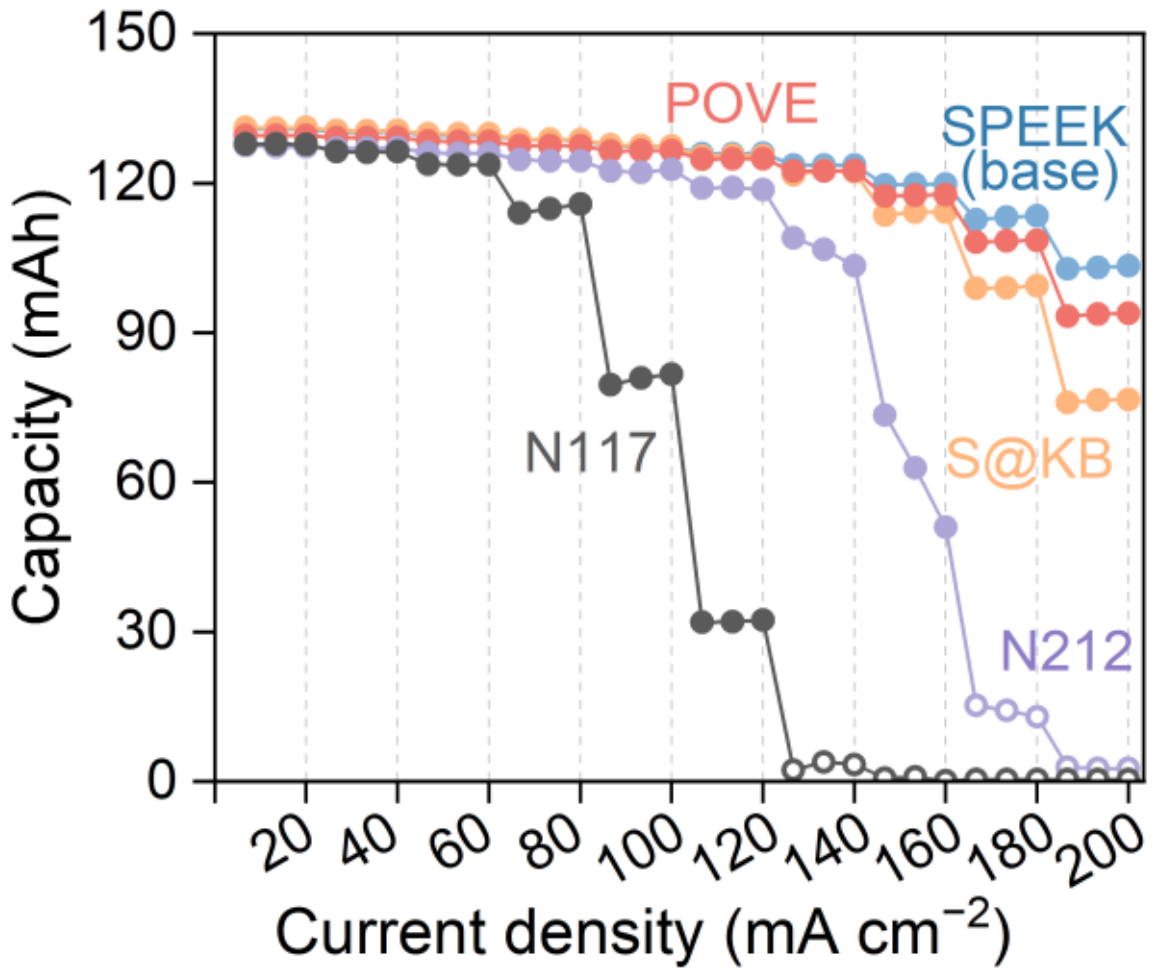

Figure S26. Discharge capacity of different IEMs under various current densities (20–200 mA cm^{-2}).

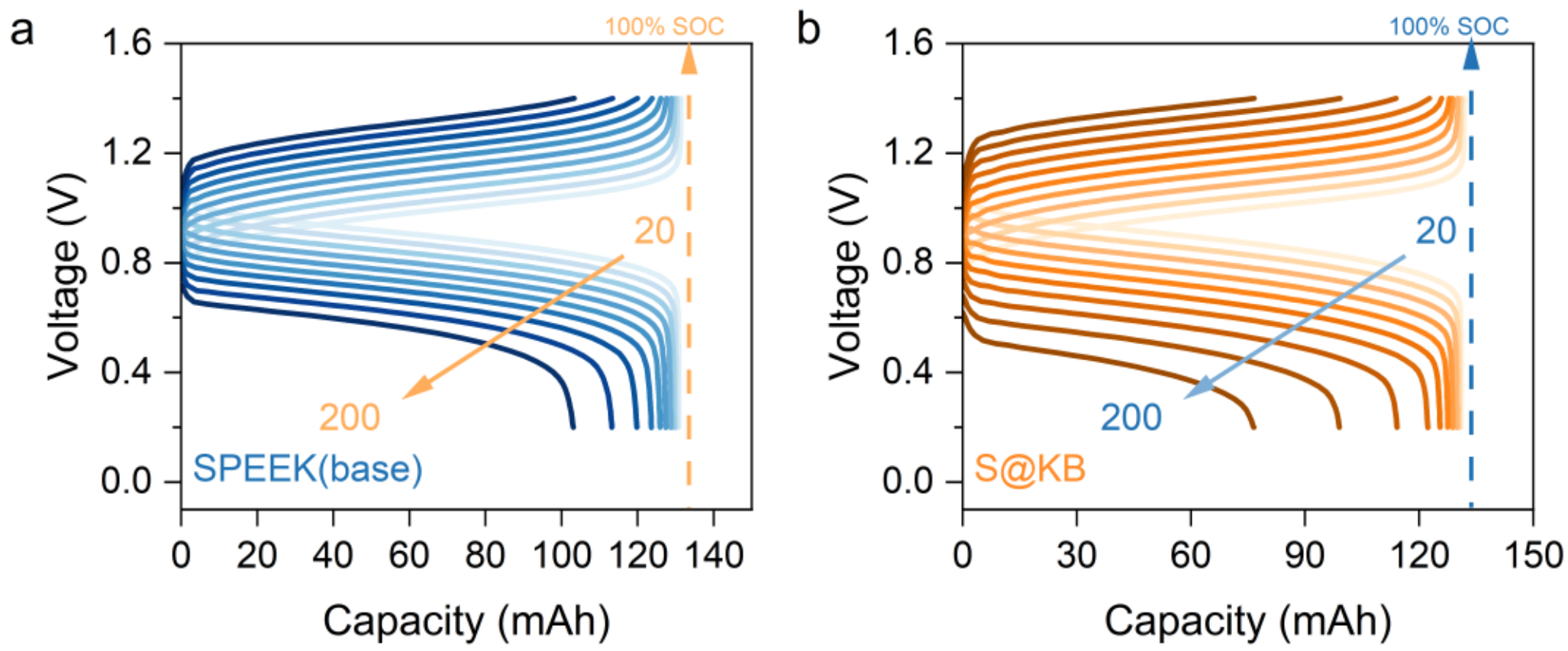

Figure S27. Voltage profiles of the S–Fe flow battery with SPEEK (base) and S@KB membrane.

(Notes: The color change indicates increasing current density from 20 mA cm^{-2} (lightest) to 200 mA cm^{-2} (darkest) with a 20 mA cm^{-2} interval.)

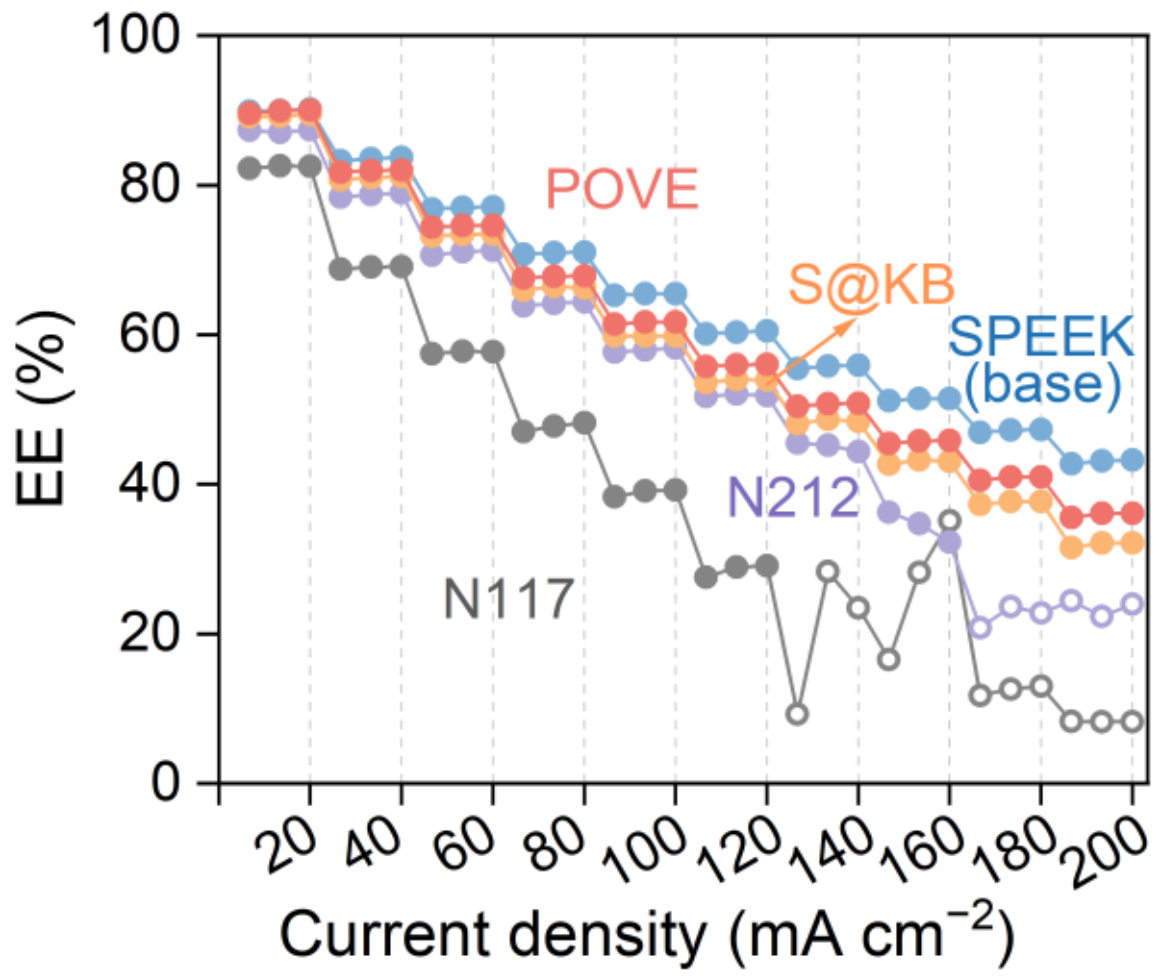

Figure S28. Energy efficiency (EE) of the S–Fe flow battery with various membranes.

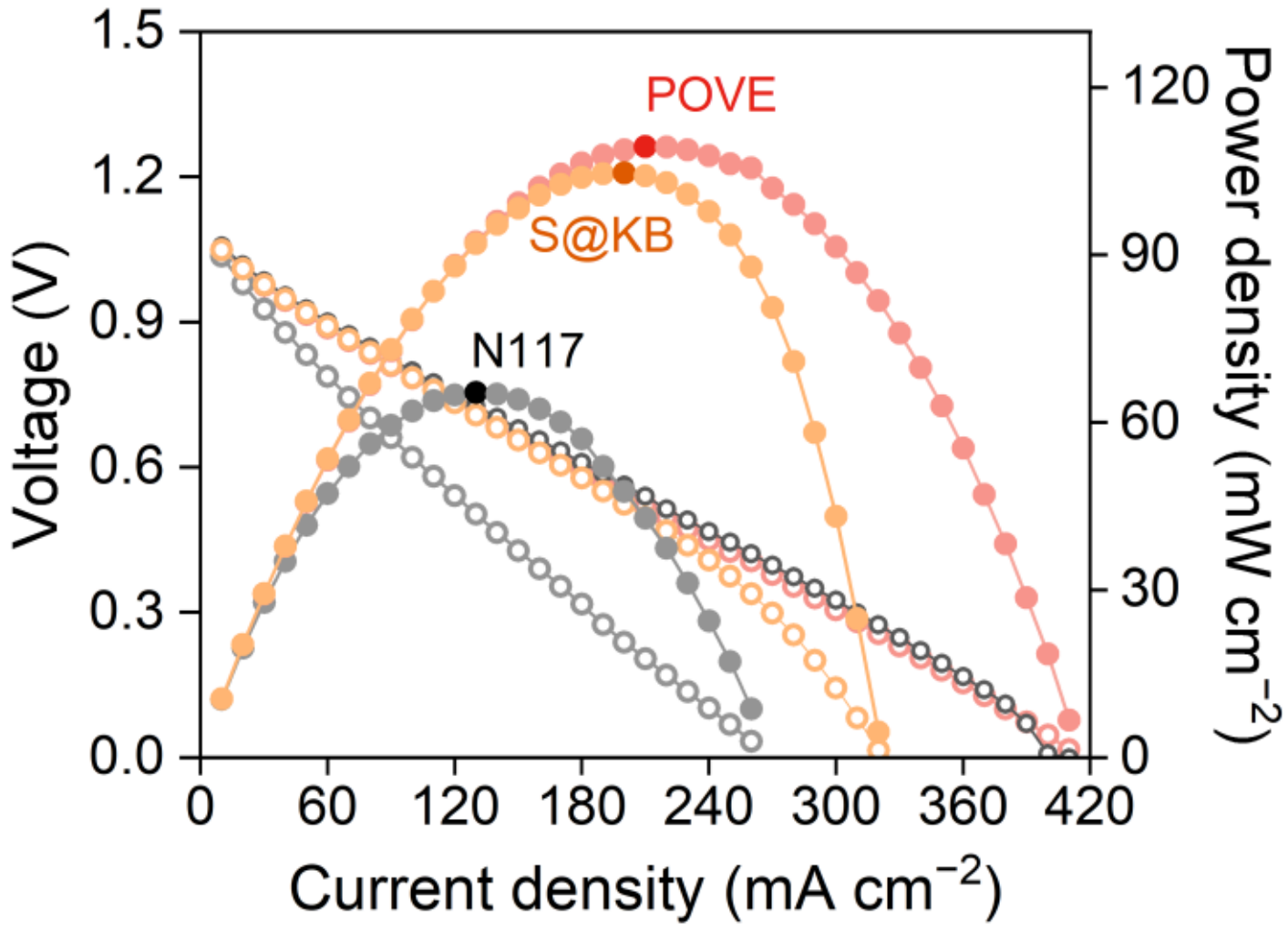

Figure S29. Output power density of the S–Fe flow battery with various IEMs.

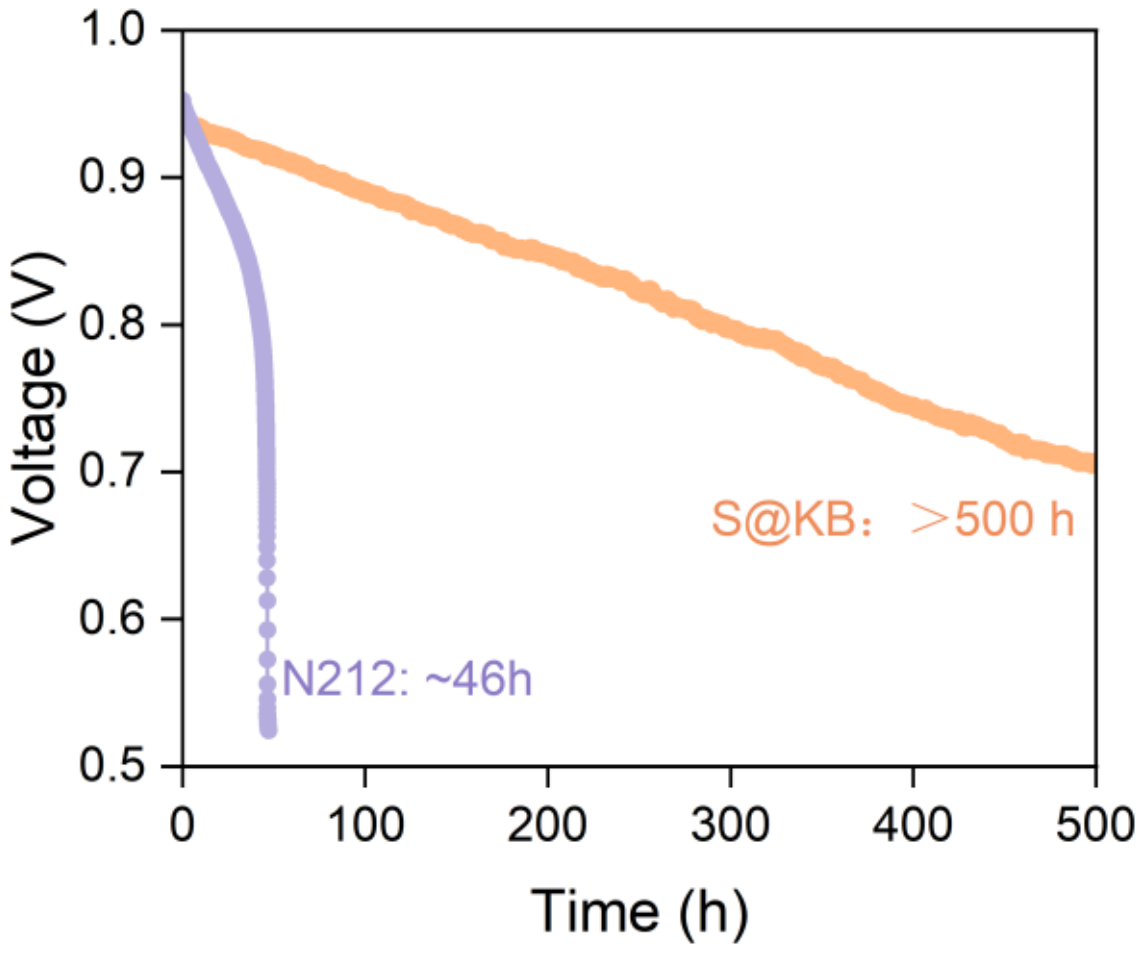

Figure S30. Open-circuit-voltage (OCV) curves of N212 and S@KB membranes.

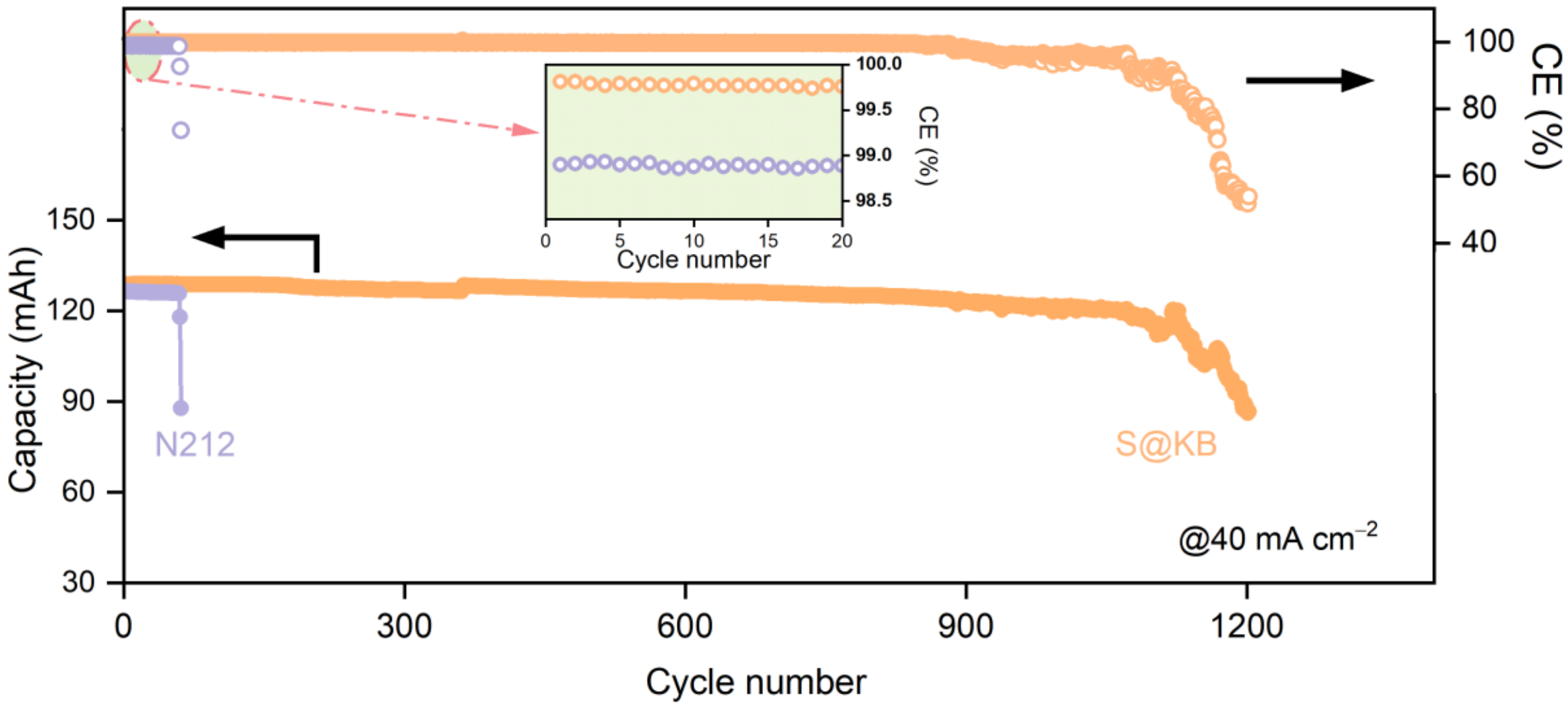

Figure S31. Cycling stability of N212 and S@KB membranes @40 mA cm^{-2}.

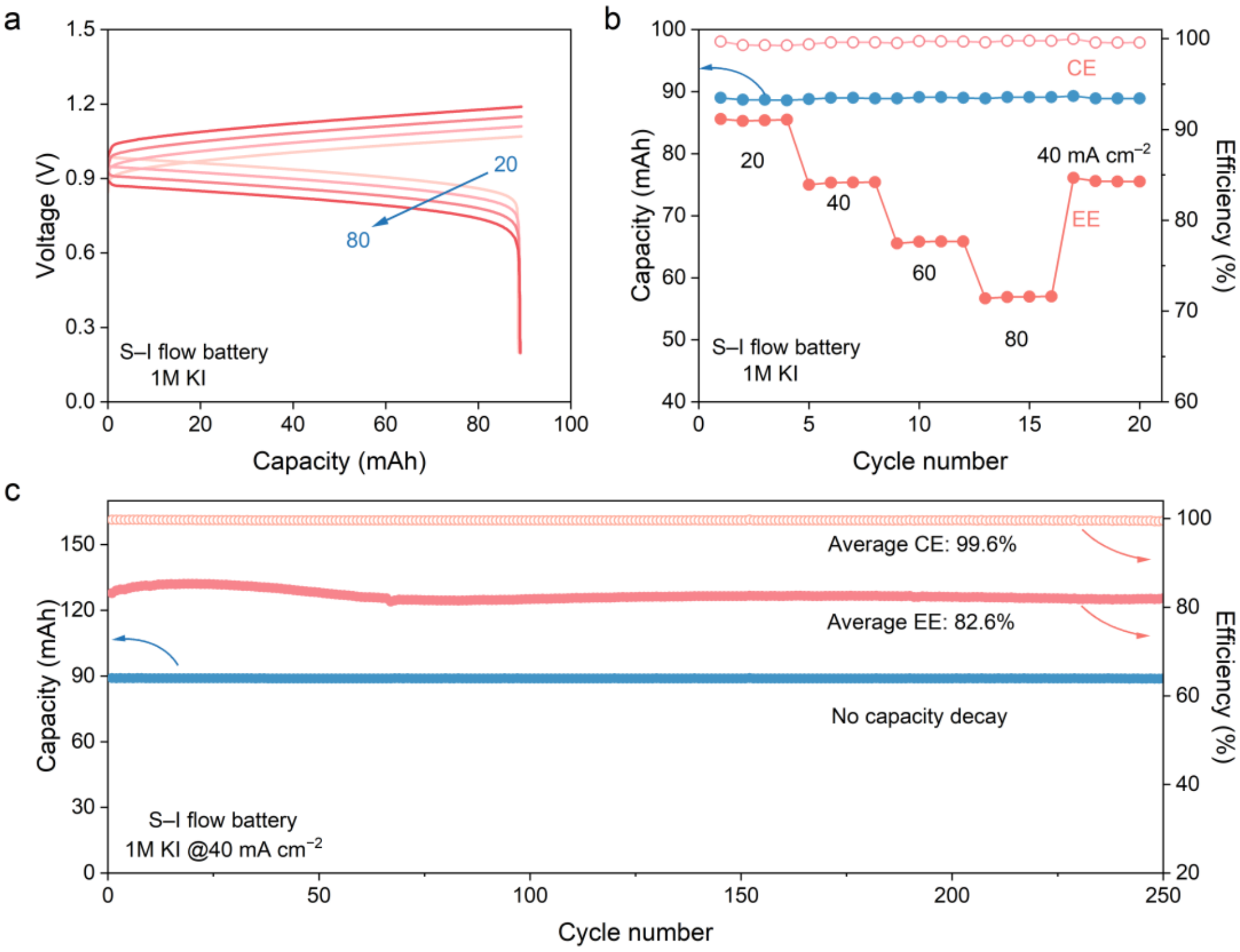

Figure S32. Electrochemical performance of polysulfide-polyiodide (S–I) flow battery assembled with the POVE membrane. a) Representative charge–discharge profiles at current densities range from 20 to 80 mA cm^{-2}; b) Discharge capacity, CE, and EE under current densities from 20 to 80 mA cm^{-2}; c) Long-term cycling performance at 40 mA cm^{-2}.

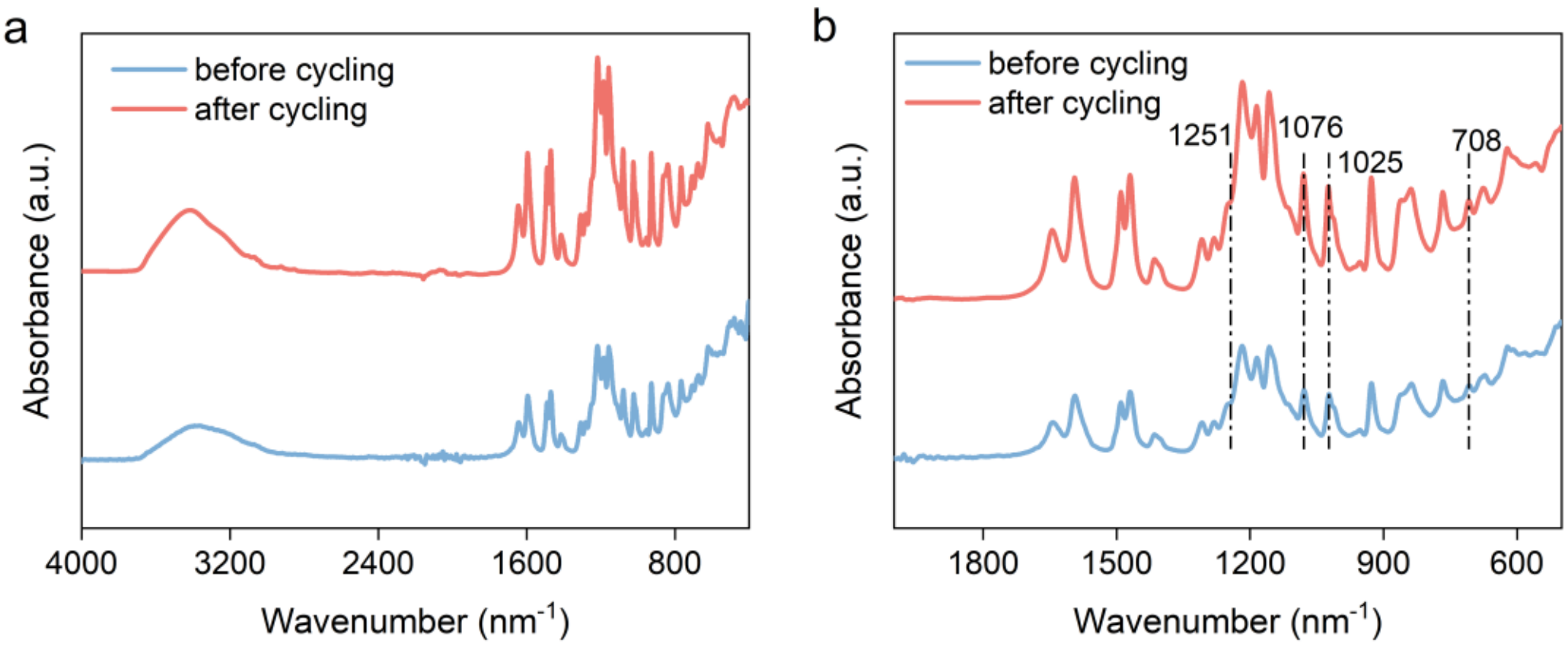

Figure S33. ATR–FITR spectra of the SPEEK (base) membrane. a) The spectra of the SPEEK membrane before and after the long-term cycling test; b) Magnified view (including characteristic absorption peaks) of SPEEK membrane before and after long-term cycling test.

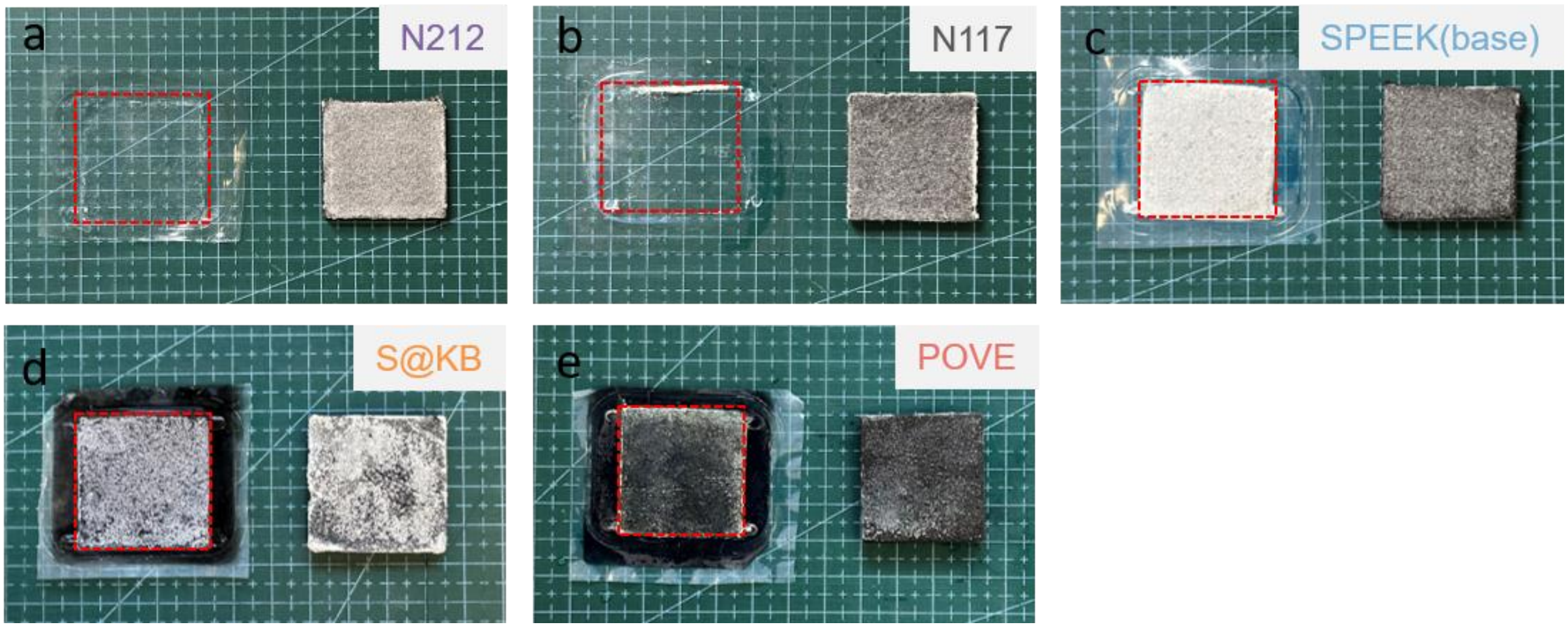

Figure S34. Digital photos of membranes and positive-side carbon felts after the cycling test. a) N212 membrane (operated for 61 cycles); b) N117 membrane (operated for 98 cycles); c) SPEEK (base) membrane (operated for 286 cycles); d) S@KB membrane (operated for ~1000 cycles); e) POVE membrane (operated over 2000 cycles).

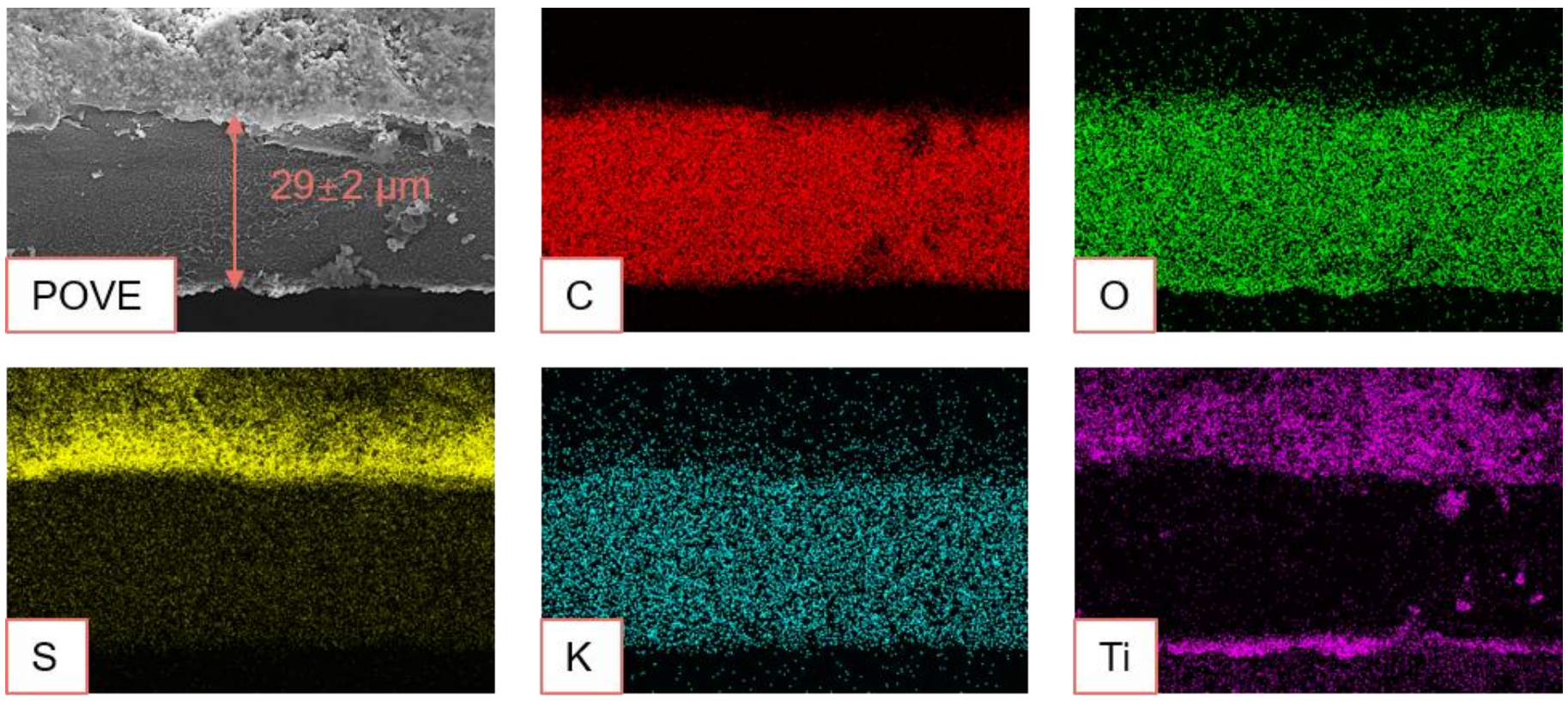

Figure S35. Energy dispersive X-ray spectroscopy (EDS) images of POVE membrane after long-term cycling test.

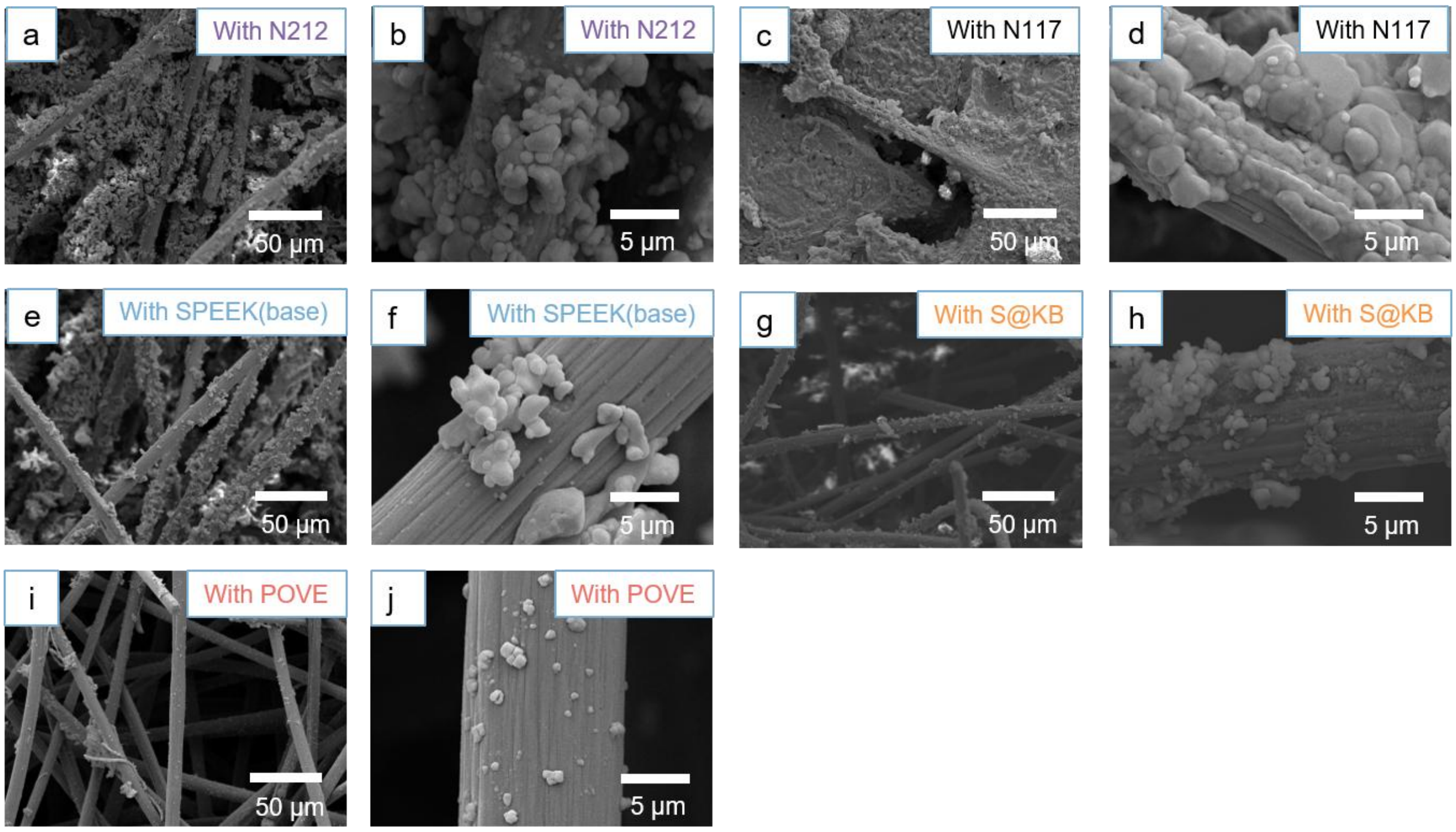

Figure S36. SEM images of positive-side carbon felts after cycling test with different IEMs. a) and b) SEM images of positive-side carbon felts after 61 cycles with the N212 membrane; c) and d) SEM images of positive-side carbon felts after 98 cycles with the N117 membrane; e) and f) SEM images of positive-side carbon felts after 286 cycles with the SPEEK (base) membrane; g) and h) SEM images of positive-side carbon felts after ~1000 cycles with the S@KB membrane; i) and j) SEM images of positive-side carbon felts after over 2000 cycles with the POVE membrane.

Table S1. Water uptake and swelling ratio of various IEMs in different solutions.

Membrane	Solution uptake (%)			Swelling ratio (%)		
	in DI water	in 1M KOH	in 1M KCl	in DI water	in 1M KOH	in 1M KCl
N117	13.5 ± 0.1	9.9 ± 0.3	8.2 ± 0.2	30.2 ± 0.1	25.9 ± 0.2	24.0 ± 0.5
N212	13.5 ± 0.4	10.1 ± 0.5	8.5 ± 0.2	30.0 ± 1.3	25.0 ± 0.2	24.8 ± 1.0
SPEEK (base)	22.2 ± 0.8	17.8 ± 0.8	16.6 ± 0.3	21.7 ± 0.6	14.8 ± 1.1	15.1 ± 0.2
S@KB	14.7 ± 0.7	13.3 ± 0.5	10.2 ± 0.6	18.2 ± 1.5	13.3 ± 1.0	12.5 ± 1.3
POVE	12.6 ± 0.5	11.2 ± 0.8	8.6 ± 0.7	17.4 ± 0.9	13.7 ± 1.1	12.6 ± 0.4

Table S2. Ionic permeability values of various membranes.

Membrane	S_4^{2-} (10^{-8} cm^2 min^{-1})	S_2^{2-} (10^{-8} cm^2 min^{-1})	$[Fe(CN)_6]^{4-}$ (10^{-8} cm^2 min^{-1})	$[Fe(CN)_6]^{3-}$ (10^{-8} cm^2 min^{-1})
N212	47.55 ± 1.68	140.31 ± 0.87	3.37 ± 0.88	6.42 ± 0.46
N117	16.65 ± 0.49	107.26 ± 1.78	4.11 ± 2.74	5.94 ± 1.79
SPEEK (base)	0.47 ± 0.05	8.60 ± 0.15	Nil	Nil
S@KB	0.34 ± 0.06	3.17 ± 0.01	Nil	Nil
POVE	0.19 ± 0.05	2.25 ± 0.15	Nil	Nil

Note: Nil represents that the absorbance spectra of corresponding ions were below the detection limit of the UV–visible spectrophotometer throughout the testing period.

Table S3. Summary of membrane ionic conductivity and selectivity.

Membrane	Ionic conductivity ($mS\ cm^{-1}$)	Polysulfide permeability ($10^{-8}\ cm^2\ min^{-1}$)	Ionic selectivity ($10^4\ S\ min\ cm^{-3}$)	Testing conditions	Ref.
SPES-30	6.25	0.303	206.27	Unspecified	[14]
SPES-50	~13.50	266.90	0.51	Unspecified	[14]
N117	8.32	9.491	8.77	Unspecified	[14]
N115	2.63	14.72[a]	1.79	Unspecified	[48]
Fumasep F-1850	0.23	0.22[a]	10.45	Unspecified	[48]
T-CEM	16.06	27.42[a]	5.86	Unspecified	[49]
PSFA	~1.00	~1.28[a]	7.81	Unspecified	[50]
P/cPIM-2%	~0.92	~0.42[a]	21.9	Unspecified	[50]
N117	~12.30	~104[b]	1.18	Argon-protected	[59]
Fumasep FAA-3-PK-130	~27.80	~249[b]	1.12	Argon-protected	[59]
N212	9.94	140.3	0.71	Argon-protected	This work
N117	10.41	107.3	0.97	Argon-protected	This work
SPEEK	6.06	8.60	7.05	Argon-protected	This work
S@KB	5.29	3.17	16.69	Argon-protected	This work
POVE	5.41	2.25	24.04	Argon-protected	This work

(Note: a. Permeability values were converted from the units; b. Permeability was calculated from polysulfide flux.)

Table S4. DFT-calculated adsorption energies, interfacial distances, and Bader charge transfer for K_2S_2 and K_2S_4 adsorption on Ti_4O_7 and KB.

Substrate	Adsorbate	Adsorption energy (eV)	Interfacial distance (Å)	Electron gain of substrate (e)
Ti_4O_7	K_2S_2	−3.25	2.57	0.87
	K_2S_4	−3.41	2.45	0.25
KB	K_2S_2	−0.71	5.92	0.37
	K_2S_4	−0.81	4.64	0.23

Table S5. The estimated cost of various ultrathin membranes.

Materials	Price (US$ m^{-2})	Calculation basis	Source
PEEK	11.91–13.21	Raw material cost	Victrex, Alibaba
sulfuric acid	0.24–0.27	Raw material cost	Alibaba
SPEEK polymer	12.15-13.48	Raw material cost, yield ratio: 92-95%	
DMF	1.62–1.81	Raw material cost	Alibaba
base-membrane conversion	0.90–1.25	Solution preparation, casting, drying, trimming, etc.	
Total for SPEEK membrane	14.67–16.54	Including raw material, material-loss assumptions and industrial conversion cost	This work
KB	0.25–0.42	Raw material cost	Alibaba, Ref. [14] and [20]
PTFE	0.04–0.06	Raw material cost	Alibaba
IPA	0.35–0.46	Raw material cost	Alibaba, Ref. [14]
Coating conversion	0.50–0.60	Slurry losses during mixing and coating, ~95% coating slurry utilization	
Total for S@KB membrane	15.81–18.08	Including raw material, material-loss assumptions and coating conversion cost	This work
Ti_4O_7	7.01–8.14	Raw material cost	Alibaba
PTFE	0.04–0.06	Raw material cost	Alibaba, Ref. [14]
IPA	0.35–0.46	Raw material cost	Alibaba
Coating conversion	0.50–0.60	Slurry losses during mixing and coating, ~95% coating slurry utilization	
Total for POVE membrane	22.57–25.80	Including raw material, material-loss assumptions and coating conversion cost	This work

Note: Gold supplier with high transaction level from www.alibaba.com. Available at https://www.alibaba.com (Accessed: 21st October 2025).

The raw material cost per unit membrane area was calculated as:

$$C_{\text{raw}} = \sum_i q_i P_i \tag{10}$$

where q_i is the areal consumption of component i, and P_i is its corresponding unit price. The preliminary total cost was then estimated as:

$$C_{\text{total}} = C_{\text{raw}} + C_{\text{base}} + C_{\text{coating}} \tag{11}$$

where C_{base} represents the conversion cost for solution preparation, membrane casting, drying, trimming, and quality control, while C_{coating} includes slurry dispersion, surface coating, drying, labor, and equipment allocation.

Table S6. Comparison of the estimated production costs of different membranes.

membrane	Price (US$ m^{-2})	Ref.
POVE	22.6–25.8	This work
S@KB	15.8–18.1	This work
SPEEK (base)	14.7–16.5	This work
SPEEK	12.3–22	[45,60]
N212	500–776	[60,61]
N117	500–2200	[20,26–28]

(Note: The different costs of various SPEEK-based IEMs stem from different preparation methods and various thicknesses of membranes.)

Table S7. Performance of recently reported polysulfide-based flow batteries.

RFB system	IEMs	IEMs Cost (US$ m^{-2})	Energy efficiency	Cycling stability	Ref.
S–Fe	CoZn-N-C coated N115	–	~60% @60 mA cm^{-2}	200 cycles (~134 h) @ 60 mA cm^{-2}	[13]
S–Fe	S30-C	12-66	77.8% @20 mA cm^{-2}	500 cycles (~1700 h) @ 20 mA cm^{-2}	[14]
S–Fe	S30-C	12-66	75% @20 mA cm^{-2}	1600 cycles (~4300 h)★ @ 20 mA cm^{-2}	[14]
S–I	CRIS	500[20]	80% @10 mA cm^{-2}	500 cycles (~2227 h) @ 10 mA cm^{-2}	[20]
S–Fe	N117	500[20]	75% @20 mA cm^{-2}	100 cycles (~750 h) @ 20 mA cm^{-2}	[43]
S–Fe	CRIS	500[20]	61% @40 mA cm^{-2}	2000 cycles (~1100 h) @ 40 mA cm^{-2}	[44]
S–I	CRIS	500[20]	59% @40 mA cm^{-2}	1300 cycles (960 h) @ 40 mA cm^{-2}	[44]
S–Fe	SPEEK-DS57	~22	~79.3% @ 20 mA cm^{-2}	230 cycles (~580 h) @ 20 mA cm^{-2}	[45]
S–I	N117+ N115	>1,000[20,61]	68% @ 10 mA cm^{-2}	70 cycles (70 h) @ 10 mA cm^{-2}	[46]
S–I	N117+ N115	>1,000[20,61]	average 82.8% @ 10 mA cm^{-2}	550 cycles (1,000 h) * @ 10 mA cm^{-2}	[47]
S–Fe	F1850	~125	~60% @ 20 mA cm^{-2}	300 cycles (1176 h) @ 20 mA cm^{-2}	[48]
S–Fe	T-CEM	–	63% @ 40 mA cm^{-2}	250 cycles (~109 h) @ 40 mA cm^{-2}	[49]
S–I	P/cPIM	~900	~80% @ 20 mA cm^{-2}	140 cycles (780 h) @ 20 mA cm^{-2}	[50]
S–Air	N117+FAA-3-PK-130	>1900[20,62]	~50% @ 1 mA cm^{-2}	80 cycles (450 h) @ 1 mA cm^{-2}	[59]
S–I	N117	500[20]	~50% @ 20 mA cm^{-2}	200 cycles (530 h)* @ 20 mA cm^{-2}	[63]
S–Ce	MFI–zeolite		~43% @ 10 mA cm^{-2}	200 cycles (~100 h) @ 10 mA cm^{-2}	[64]
S–I	N117+ N115	>1,000[20,59]	70% @20 mA cm^{-2}	50 cycles (~130 h) @20 mA cm^{-2}	[65]
S–Fe	POVE	22.6–25.8	82% @40 mA cm^{-2}	2000 cycles (> 1100 h) @40 mA cm^{-2}	This work

(Note: * represents the electrolyte refreshment 3 times, and ★ represents the recovery process according to the article.)

Supplementary Note 1 | Mechanical, thermal, and chemical properties of the various IEMs.

The mechanical properties of various IEMs were evaluated (Figure S13). Despite their ultrathin nature, all hydrocarbon-based membranes exhibited comparable tensile strengths to those of Nafion series membranes. In terms of elongation at break, the pristine SPEEK (base) membrane showed a similar value to that of N212, indicating a well-balanced combination of toughness and rigidity. Incorporation of functional materials further enhanced the rigidity of the decorated membranes. The POVE membrane was also tested under continuous water flushing in a flow battery configuration for 384 h (Figure S14). The flow rates ranged from 20–80 mL min^{-1}, covering the electrolyte flow rate of 50 mL min^{-1} used during normal battery operation and extending to a more demanding flow condition. No visible Ti_4O_7 residuals were observed in the water reservoir, indicating that the functional layer remained firmly attached to the SPEEK substrate under prolonged hydrodynamic shear.

Thermogravimetric analysis (TGA) was conducted to assess the thermal stability of membranes (Figure S15). In contrast to N117 and N212 membranes, which displayed a single rapid weight-loss zone, the SPEEK, S@KB, and POVE membranes exhibited two distinct decomposition stages. The first stage (300–380 °C) corresponds to the detachment of sulfonic acid groups, and the second stage is associated with the degradation of the hydrocarbon backbones. These results confirmed that the prepared membranes maintained structural stability and integrity within the temperature range relevant to flow battery operation.

The chemical stability of different IEMs was tested by immersing them in posolytes and negolytes for 60 days, respectively (Figure S16). ATR–FTIR spectra showed that the characteristic vibrational peaks of both perfluorosulfonic acid Nafion and hydrocarbon-based SPEEK membranes remained essentially unchanged, indicating their high chemical stability under operating conditions.

Supplementary Note 2 | Density functional theory (DFT) calculation reveals adsorption behaviors of polysulfides on Ti_4O_7 and KB.

Density functional theory (DFT) calculations revealed the theoretical adsorption energies of −3.25 eV (K_2S_2) and −3.41 eV (K_2S_4) on Ti_4O_7, significantly stronger than those on KB (−0.71 eV for K_2S_2 and −0.81 eV for K_2S_4; **Figure 3**b and Figure S17). The average distances between the S atoms of the adsorbed K_2S_2 and K_2S_4 species and the KB surface were 5.92 and 4.64 Å, respectively. These relatively large distances indicated that the interactions between KB and the polysulfides were predominantly weak and nonbonding. In contrast, direct Ti–S bonds were formed in the adsorption of K_2S_2 and K_2S_4 on the Ti_4O_7 surface, with the average Ti–S bond lengths of 2.57 and 2.45 Å, respectively. The considerably shorter Ti–S distances supported the chemisorption of polysulfides on Ti_4O_7. The Bader charge analysis further revealed electron transfer from the adsorbed polysulfide species to both substrates. Upon K_2S_2 adsorption, the Ti_4O_7 gained approximately 0.87 e, substantially larger than that of KB substrates (0.37 e); for K_2S_4 adsorption, the corresponding electron gains of the Ti_4O_7 and KB substrates were approximately 0.25 e and 0.23 e, respectively. The results indicated stronger interfacial electronic interactions of polysulfides with Ti_4O_7 than with KB. The more negative adsorption energies (**Figure 3**b), the markedly shorter Ti–S distances, and the Bader charge transfer results (Table S4) collectively demonstrate that Ti_4O_7 possesses a stronger affinity toward polysulfides than KB.

Supplementary Note 3 | theoretical calculations analyze the ion-specific gating strategy.

The fundamental mechanism relies on the Ti_4O_7 functional layer located at the membrane-electrolyte interface, where it selectively captures active polysulfide species through strong chemisorption while hindering the transport of K^+ ions. We performed diffusion energy barrier calculations and *ab initio* molecular dynamics (AIMD) simulations under aqueous conditions to elucidate the interfacial ion-specific gating mechanism of Ti_4O_7.

First, we calculated the diffusion energy barriers of K and representative polysulfide on the Ti_4O_7 surface from a kinetic perspective (Figure S21). We found that the diffusion barrier of K atom on Ti_4O_7 surface was only around 0.18 eV. This indicated that although K may interact with oxygen sites on the Ti_4O_7 surface from the adsorption results, it can still migrate along the interface through a relatively low-energy pathway. In contrast, the diffusion barrier of polysulfide atoms on Ti_4O_7 was approximately 2.2 eV, which was significantly higher than that of K. The high migration barrier kinetically restricted further diffusion of polysulfide species on the Ti_4O_7 surface and promoted their interfacial adsorption. Therefore, the diffusion barriers demonstrated that Ti_4O_7 favored anchor polysulfides while maintaining efficient K transport.

To further simulate the realistic electrolyte environment, AIMD simulations were performed under aqueous conditions. An interfacial model consisting of Ti_4O_7, two K^+ ions, one S_2^{2-}, and multiple water molecules was constructed, and the dynamic evolution was simulated at 298 K.[66] In the initial configuration, the K^+ ions and S_2^{2-} were placed at the same distance from the Ti_4O_7 surface to investigate their dynamic behaviors. The AIMD trajectories were statistically analyzed by tracking the evolution of Ti–S and K–O surface distances over time (Figure S22). The Ti–S distance rapidly decreased at the beginning of the simulation and eventually stabilized within a certain range. This indicates that the polysulfide spontaneously approached and adsorbed onto the Ti_4O_7 surface, forming stable Ti–S interactions. While the K–O (Ti_4O_7) distance gradually increased with fluctuations. Besides, the vertical distance between the K^+ and S_2^{2-} from the Ti_4O_7 surface further verified the results (Figure S23). With increasing simulation time, the S atoms remained close to the Ti_4O_7 surface, with the vertical distance (ΔZ) around 1.6 Å, consistent with the stable chemical interaction between polysulfide and Ti_4O_7. While the distance of K^+ ion from the surface increased, oscillating around 2.6 Å, suggesting that K^+ ion experienced negligible interfacial interactions with Ti_4O_7. These results demonstrated the stable

preferential interaction of Ti_4O_7 with polysulfides under aqueous environment while preserving the hydrated transport state of K^+ ions.

In summary, through theoretical kinetics analysis (diffusion barriers) and AIMD simulations, we demonstrate that the Ti_4O_7 interface possesses an ion-specific gating function. The active Ti sites on Ti_4O_7 selectively immobilize polysulfides through strong Ti–S chemisorption, whereas K ions maintain rapid transport due to their low migration barrier and stable hydration structure. These results further support the interfacial ion-specific gating mechanism proposed in this work.

Supplementary Note 4 | Characterization of different membranes (after long-term cycling test)

We characterized the positive-side carbon felts and IEMs after the long-term cycling test. In the ATR–FITR spectra of the SPEEK (base) membrane, the characteristic peaks remained unchanged after prolonged cycling test, indicating preserved functional groups and structural integrity of the membrane substrate (Figure S33). As shown in Figure S34, substantially non-conductive sulfur accumulated at the membrane–electrode interface in the S–Fe flow batteries employing N117 and N212 membranes, even though these batteries operated for less than 100 cycles, further indicating severe polysulfide crossover. In contrast, among all hydrocarbon-based SPEEK membranes, the POVE membrane achieved the longest cycling life while exhibiting the least sulfur deposition. Besides, the POVE membrane maintained a compact structure without interfacial separation or surface peeling (Figure S35). Corresponding energy-dispersive X-ray spectroscopy (EDS) images confirmed the Ti-containing layer remained evenly distributed over the SPEEK substrate after cycling. These results collectively demonstrate the robust adhesion of functional layer under prolonged hydrodynamic and electrochemical operation. SEM images of positive-side carbon felts assembled with different membranes are presented in Figure S36. The amount of sulfur deposited on the electrodes follows the order of POVE < S@KB < SPEEK (base) < N117 < N212, which is consistent with the polysulfide permeability trends established earlier in both static H-cell permeation tests and flow mode OCV measurements. These results further validate the exceptional polysulfide-blocking ability of the prepared POVE membrane.

Reference

[13] B. Chen, H. Huang, J. Lin, et al., "Doping Engineering of M-N-C Electrocatalyst Based Membrane-Electrode Assembly for High-Performance Aqueous Polysulfides Redox Flow Batteries," *Advanced Science* **10**, (2023): 2206949, https://doi.org/10.1002/advs.202206949.

[14] F. Wang, S. Luo, J. Lei, et al., "Nonfluorinated Membrane with a Decentralized Ion-Transport Network Enables Efficient and Sustainable Polysulfide Redox Flow Batteries," *Science Advances* **11**, (2025): eaea0032, https://doi.org/10.1126/sciadv.aea0032.

[20] Z. Li and Y.-C. Lu, "Polysulfide-Based Redox Flow Batteries with Long Life and Low Levelized Cost Enabled by Charge-Reinforced Ion-Selective Membranes," *Nature Energy* **6** (2021): 517–528, https://doi.org/10.1038/s41560-021-00804-x.

[26] M. Oliot, S. Galier, H. Roux de Balmann, and A. Bergel, "Ion Transport in Microbial Fuel Cells: Key Roles, Theory and Critical Review," *Applied Energy* **183**, (2016): 1682–1704, https://doi.org/10.1016/j.apenergy.2016.09.043.

[27] H. Prifti, A. Parasuraman, S. Winardi, T. M. Lim, and M. Skyllas-Kazacos, "Membranes for Redox Flow Battery Applications," *Membranes* **2**, (2012): 275-306, https://doi.org/10.3390/membranes2020275.

[28] O. S. J. Elham, S. K. Kamarudin, N. Shaari, et al., "Development of Low-Cost Nafion-Lignin Composite Conductive Membranes for Application in Direct Methanol Fuel Cells," *Journal of Environmental Chemical Engineering* **12**, (2024): 111514, https://doi.org/10.1016/j.jece.2023.111514.

[43] X. Wei, G.-G. Xia, B. Kirby, et al., "An Aqueous Redox Flow Battery Based on Neutral Alkali Metal Ferri/ferrocyanide and Polysulfide Electrolytes," J*ournal of the Electrochemical Society* **163**, (2016): A5150-A5153, https://doi.org/10.1149/2.0221601jes.

[44] J. Lei, Y. Zhang, Y. Yao, et al., "An Active and Durable Molecular Catalyst for Aqueous Polysulfide-Based Redox Flow Batteries," *Nature Energy* **8**, (2023): 1355–1364, https://doi.org/10.1038/s41560-023-01370-0.

[45] X. Lou, H. Fu, J. Xu, et al., "Cost-Effective Membrane and Advanced Electrode for Stable Polysulfide-Ferricyanide Flow Battery," *Energy Material Advances* **2022**, (2022): 9865618, https://doi.org/10.34133/2022/9865618.

[46] Z. Li, G. Weng, Q. Zou, et al., "A High-Energy and Low-Cost Polysulfide/Iodide Redox Flow Battery," *Nano Energy* **30**, (2016): 283-292, https://doi.org/10.1016/j.nanoen.2016.09.043.

[47] D. Ma, B. Hu, W. Wu, et al., "Highly Active Nanostructured CoS_2/CoS Heterojunction Electrocatalysts for Aqueous Polysulfide/Iodide Redox Flow Batteries," *Nature Communications* **10**, (2019): 3367, https://doi.org/10.1038/s41467-019-11176-y.

[48] M. S. Khabbaz, A. Thakur, D. Maity, et al., "A Polysulfide/Ferricyanide Redox Flow Battery with Extended Cycling," *Journal of Materials Chemistry A* **13** (2025): 29610–29620. https://doi.org/10.1039/d5ta05404d.

[49] S. Sreenath, Nayanthara P. S., C. M. Pawar, et al., "An Aqueous Polysulfide Redox Flow Battery with a Semi-Fluorinated Cation Exchange Membrane," *Energy Advances* **3**, (2024): 203–214. https://doi.org/10.1039/d3ya00509g.

[50] Q. Hou, H. Cao, Y. Xia, et. al., "Rigid Framework-Constrained Membranes: A Key to Long-Life Polysulfide/Polyiodide Redox Flow Batteries," *ACS Applied Materials & Interfaces* **17**, (2025): 40508–40516. https://doi.org/10.1021/acsami.5c07898.

[51] G. Kresse and D. Joubert, "From Ultrasoft Pseudopotentials to the Projector Augmented-Wave Method," *Physical Review B* **59**, (1999): 1758, https://doi.org/10.1103/PhysRevB.59.1758.

[52] S. Grimme, "Semiempirical GGA-Type Density Functional Constructed with a Long-Range Dispersion Correction," *Journal of Computational Chemistry* **27**, (2006): 1787-1799, https://doi.org/10.1002/jcc.20495.

[53] G. Kresse and J. Furthmüller, "Efficiency of Ab-Initio Total Energy Calculations for Metals and Semiconductors Using a Plane-Wave Basis Set," *Computational Materials Science* **6**, (1996): 15-50, https://doi.org/10.1016/0927-0256(96)00008-0.

[54] S. Grimme, J. Antony, S. Ehrlich,and H. Krieg, "A Consistent and Accurate *Ab Initio* Parametrization of Density Functional Dispersion Correction (DFT-D) for the 94 Elements H-Pu," *Journal of Chemical Physics* **132**, (2010): 154104, https://doi.org/10.1063/1.3382344.

[55] Y. Xiong, Y. Wang, N. Ma, et al., "First Principles Study of B_7N_5 as a High Capacity Electrode Material for K-Ion Batteries," *Physical Chemistry Chemical Physics* **25**, (2023): 24303-24312, https://doi.org/10.1039/D3CP02391E.

[56] G. Henkelman, B. P. Uberuaga, Hannes Jónsson, "A Climbing Image Nudged Elastic Band Method for Finding Saddle Points and Minimum Energy Paths," *Journal of Chemical Physics* **113**,

(2000): 9901–9904, https://doi.org/10.1063/1.1329672.
[57] J. Lei, Y. Zhang, W. Wu, et al., "Bio-Inspired Relay Catalysis for Aqueous Redox Flow Batteries," *Nature Communications* **17**, (2026) 6882, https://doi.org/10.1038/s41467-026-73670-4.
[58] R. Tan, A. Wang, R. Malpass-Evans, et al., "Hydrophilic Microporous Membranes for Selective Ion Separation and Flow-Battery Energy Storage," *Nature Materials* **19**, (2020): 195–202, https://doi.org/10.1038/s41563-019-0536-8.
[59] Y. Xia, M. Ouyang, V. Yufit, et al., "A Cost-Effective Alkaline Polysulfide-Air Redox Flow Battery Enabled by a Dual-Membrane Cell Architecture," *Nature Communications* **13**, (2022): 2388, https://doi.org/10.1038/s41467-022-30044-w.
[60] Z. Yuan, L. Liang, Q. Dai, et al., "Low-Cost Hydrocarbon Membrane Enables Commercial-Scale Flow batteries for Long-Duration Energy Storage," *Joule* **6**, (2022): 884-905, https://doi.org/10.1016/j.joule.2022.02.016.
[61] J. Xi, B. Jiang, L. Yu, and L. Liu, "Membrane Evaluation for Vanadium Flow Batteries in a Temperature Range of −20–50 °C," *Journal of Membrane Science* **522**, (2017): 45-55, https://doi.org/10.1016/j.memsci.2016.09.012.
[62] J. Li, J. Li, Z. Cao, et al., "Enhanced OH^- Conductivity and Stability of Polybenzimidazole Membranes for Electrocatalytic CO_2 Reduction via Grafting and Crosslinking Strategies," *Journal of Membrane Science* **685**, (2023): 121985, https://doi.org/10.1016/j.memsci.2023.121985.
[63] L. Su, A. F. Badel, C. Cao, et al., "Toward an Inexpensive Aqueous Polysulfide–Polyiodide Redox Flow Battery," *Industrial & Engineering Chemistry Research* **56**, (2017): 9783–9792, https://doi.org/10.1021/acs.iecr.7b01476.
[64] P. Silambarasan, A. G. Ramu, M. Govarthanan, et al., "Cerium-Polysulfide Redox Flow Battery with Possible High Energy Density Enabled by MFI-Zeolite Membrane working with Acid-Base Electrolytes," *Chemosphere* **291**, (2022): 132680, https://doi.org/10.1016/j.chemosphere.2021.132680.
[65] Y. Zhu, K. He, T. T. Tsega, et al., "Cu_2CoGeS_4 Nanocrystals for High Performance Aqueous Polysulfide/Iodide Redox Flow Batteries: Enhanced Selectively towards the Electrocatalytic Conversion of Polysulfides," *Sustainable Energy & Fuels* **4**, (2020): 2892-2899, https://doi.org/10.1039/C9SE01201J.

[66] M. Wang, T. Sadhukhan, N. H. C. Lewis, et al., “Anomalously Enhanced Ion Transport and Uptake in Functionalized Angstrom-Scale Two-Dimensional Channels,” *Proceedings of the National Academy of Sciences* **121**, (2024): e2313616121, https://doi.org/10.1073/pnas.2313616121.
